# Quantitative dual-isotope preclinical SPECT/CT imaging and biodistribution of the mercury-197m/g theranostic pair with [^197m/g^Hg]HgCl_2_ and a [^197m/g^Hg]Hg-tetrathiol complex as a platform for radiopharmaceutical development

**DOI:** 10.1186/s41181-025-00391-2

**Published:** 2025-10-14

**Authors:** Parmissa Randhawa, Cristina Rodríguez-Rodríguez, Helena Koniar, Patrick R. W. J. Davey, Shaohuang Chen, Valery Radchenko, Caterina F. Ramogida

**Affiliations:** 1 Department of Chemistry, Simon Fraser University, 8888 University Drive, Burnaby, BC V5A 1S6 Canada; 2 Life Sciences, TRIUMF, 4004 Wesbrook Mall, Vancouver, BC V6T 2A3 Canada; 3 Faculty of Pharmaceutical Sciences, University of British Columbia, 2405 Wesbrook Mall, Vancouver, BC V6T 1Z3 Canada; 4 Department of Physics and Astronomy, University of British Columbia, 6224 Agronomy Road, Vancouver, BC V6T 1Z1 Canada; 5 Department of Chemistry, University of British Columbia, 2036 Main Mall, Vancouver, BC V6T 1Z1 Canada

**Keywords:** Mercury-197, SPECT, Theranostics, Tetrathiol, Chelators, Hg^2+^, Phantom imaging

## Abstract

**Background:**

Mercury-197m (^197m^Hg, t_1/2_ = 23.8 h) and mercury-197g (^197g^Hg, t_1/2_ = 64.14 h) possess favorable nuclear properties for imaging and targeted therapy, but the development of suitable chelators for mercury-based radiopharmaceuticals remains underexplored. Additionally, accurate imaging and quantification of mercury isotopes, particularly in dual-isotope formats, require tools that account for their complex decay schemes. Phantom imaging studies are essential for validating spatial resolution, quantitative accuracy, and isotope-specific calibration prior to in vivo application. In this study, we investigated the commercially available ligand H_4_Tetrathiol for chelation of [^197m/g^Hg]Hg^2+^ and developed a robust imaging and quantification pipeline to support the use of these nuclear isomers in preclinical imaging.

**Results:**

Radiolabeling of H_4_Tetrathiol yielded exceptionally efficient complexation, achieving the lowest ligand-to-metal ratio reported for radio-mercury. The resulting [^197m/g^Hg]Hg^2+^-complex demonstrated high in vitro stability in the presence of serum proteins, glutathione, and competing biologically relevant metal ions, though it exhibited kinetic lability when challenged with excess HgCl₂. In vivo biodistribution studies in mice showed a distinct pharmacokinetic profile from unchelated [^197m/g^Hg]HgCl₂, suggesting in vivo complex stability. Phantom imaging studies with a high sensitivity collimator demonstrated submillimeter resolution (≥ 1.1 mm) for both ^197g^Hg and ^197m^Hg, with decay behavior consistent with known half-lives. To facilitate accurate quantification, we developed *HgQuant*, a Python-based tool for isotope-specific calibration, Bateman decay correction, and automated dual-isotope analysis. This tool enabled reproducible, time-resolved quantification in both phantom and in vivo settings.

**Conclusions:**

These results establish Tetrathiol as a promising scaffold for mercury-based theranostics, offering efficient radiolabeling and in vivo stability. The integration of high-resolution imaging and *HgQuant*-based quantification of each isomer establishes a comprehensive framework for advancing [^197m/g^Hg]Hg radiopharmaceutical development.

**Supplementary Information:**

The online version contains supplementary material available at 10.1186/s41181-025-00391-2.

## Background

There is growing interest in advancing theranostic radiopharmaceuticals in nuclear medicine. These pharmaceuticals incorporate chemically similar radionuclides into the same molecular scaffold, enabling both diagnostic imaging and therapeutic applications in molecular oncology. Since they share the same molecular scaffold, the biodistribution of the diagnostic and therapeutic pharmaceuticals should be identical (Miller et al. 2022; Carbo-Bague and Ramogida 2021). This consistency allows physicians to accurately monitor the distribution and the therapeutic efficacy of the radiopharmaceutical via imaging, paving the way for more personalized treatment options (Miller et al. 2022). Radionuclides that emit Meitner-Auger electrons (MAEs) hold great potential for radiopharmaceutical therapy, with high linear energy transfer (LET, 4–26 keV/µm) and short pathlengths in tissue (1–20 µm) this particulate radiation can treat disease at the cellular level. Moreover, many radionuclides that emit MAEs can be produced on small medical cyclotrons accessible in many nuclear medicine hospitals (Carbo-Bague and Ramogida 2021; Randhawa et al. 2021; Filosofov et al. 2021; Boswell and Brechbiel 2005; Aghevlian et al. 2017; Ku et al. 2019).

**Fig. 1:**
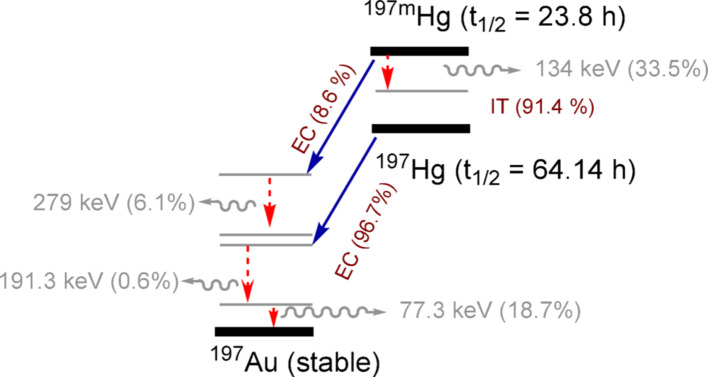
Simplified decay scheme for ^197m^Hg and ^197g^Hg towards stable ^197^Au (transitions resulting in pertinent photon emissions from each isomer highlighted with red arrows); decay radiation referenced from IAEA live chart of nuclides. EC = electron capture; IT = isomeric transition

In recent years, the isomers mercury-197m (^197m^Hg, half-life, t_1/2_ = 23.8 h) and ^197g^Hg (t_1/2_ = 64.14 h) have garnered interest in nuclear medicine due to their simultaneous release of a highly potent dose of MAEs (average no./decay = 19.4 and 23.2; average energy/decay = 13.5 and 16.1 keV, for m and g states, respectively) and conversion electrons (CEs; average no./decay = 1.6 and 0.8; average energy/decay = 203.5 and 54.15 keV, for m and g states, respectively) as well as gamma and x-ray photons compatible with single-photon emission computed tomography (SPECT) imaging, making them a chemically-matched theranostic pair (Fig. 1) (Randhawa et al. 2021; Cai et al. 2023). The use of isotopes of the same element for both imaging and therapy ensures identical chemical behaviour, allowing precise correlation of imaging with therapeutic dose for accurate dosimetry. The emission of short-ranged MAEs and higher-energy CEs enables treatment of micro-metastases and small lesions within a few millimeters in diameter, respectively. Theoretically, ^197m^Hg and ^197g^Hg deliver 2–3 times more dose to a single targeted cell than ^111^In, a clinically used SPECT radionuclide previously studied for targeted MAE therapy (Cai et al. 2023). Moreover, the metastable and ground states of mercury-197 emit photons at 134 keV (*I* = 33.5%) and 279 keV (*I* = 6.1%), respectively (Fig. 1), which are suitable for SPECT imaging (Randhawa et al. 2021).

To realize the potential of this isomeric pair in radiopharmaceutical applications, several key developments are required:

- the radionuclides must be produced and isolated at high specific activity;
- suitable bifunctional chelators (BFCs) must be identified to stably bind the “chemically-soft” [^197m/g^Hg]Hg^2+^ ion to disease targeting vectors; and
- the theranostic potential (i.e., therapeutic and imaging capabilities) of the isomers must be validated.

With regard to production, high specific activity, no-carrier added ^197m/g^Hg can be produced on a medical cyclotron by irradiating monoisotopic gold-197 via the ^197^Au(p,n)^197m/g^Hg nuclear reaction, which generates both isomers in approximately equal proportions at end of bombardment (Chen et al. 2023; Walther et al. 2015; Despotopulos and Kmak 2021; Despotopulos et al. 2018). Because gold is naturally monoisotopic, isotopic enrichment of the target material is unnecessary, significantly reducing production costs. The production of both isomers can be achieved by irradiating Au targets with proton energies < 15 MeV, with maximum cross sections around 12 MeV (Červenák and Lebeda 2019),cyclotrons with these proton energies are common in nuclear medicine hospitals, increasing the potential for widespread availability of ^197m/g^Hg.

**Fig. 2:**
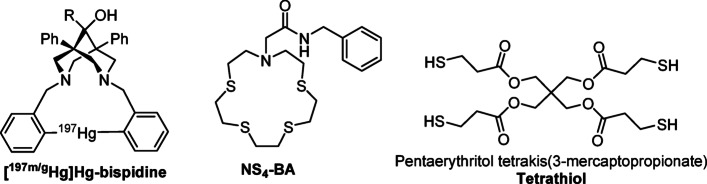
Select chelators developed for [^197m/g^Hg]Hg-radiopharmaceuticals, Hg-bispidine developed by Gilpen et al., (left); thio-ether NS_4_-BA developed by our group (centre); and the commercial chelator, pentaerythritol tetrakis(3-mercaptopropionate) (right), referred herein as Tetrathiol, investigated in this study

Chelators capable of forming stable and inert complexes with radiomercury are limited or currently unavailable. Previous work by Gilpin et al. introduced a cyclic organometallic bispidine compound (Fig. 1), which demonstrated high radiolabeling yields with radiomercury (Gilpin et al. 2021). Our group has focused on applying thioether-based macrocyclic ligands, originally developed for mercury sensing, for use in [^197m/g^Hg]Hg-radiopharmaceutical development (Randhawa et al. 2024, 2023; Tosato et al. 2024). Notably, *N*-benzyl-2-(1,4,7,10-tetrathia-13-azacyclopentadecan-13-yl)acetamide (NS_4_-BA) (Fig. 2) exhibited favourable chelation properties, achieving quantitative complexation of [^197m/g^Hg]Hg^2+^ within 60 min at both 80 °C and 37 °C, using a ligand concentration of 10^–4^ M. The resulting radiometal-complex demonstrated promising stability when challenged with human serum and glutathione (Randhawa et al. 2023).

Although no experimental therapeutic investigations have been reported to date, a few reports into ^197m/g^Hg SPECT imaging have emerged (Gilpin et al. 2021; Freudenberg et al. 2018). Most notably, Freudenberg et al. demonstrated the feasibility of ^197m/g^Hg for molecular imaging using a clinical Philips BrightView SPECT camera, supported by phantom experiments and Monte Carlo simulations (Freudenberg et al. 2018). Gilpin et al. also reported preclinical in vivo SPECT/CT imaging of [^197m/g^Hg]HgCl_2_ and their radiolabeled bispidine complex in mice (Gilpin et al. 2021). However, the metastable and ground-state signals were not clearly deconvoluted or resolved, and decay correction was performed manually using a reference source.

This manuscript presents a comprehensive approach to advance both the chelation chemistry and quantitative SPECT imaging capabilities of ^197m/g^Hg in preclinical models. Deviating from our former thioether chelators, we investigated the ^197m/g^Hg radiolabeling of a commercial pentaerythritol tetrakis(3-mercaptopropionate) acyclic ligand (H_4_Tetrathiol, Fig. 1). This scaffold serves as a model for thiol-rich chelators which are well known to form stable complexes with Hg^2+^ in vivo (Oram et al. 1996; Stricks and Kolthoff 1953; Leiva-Presa et al. 2004; Patrick 2002; Kromidas et al. 1990; Bjørklund et al. 2019; Aaseth et al. 2018). The resulting ^197m/g^Hg-tetrathiol complex was evaluated for in vitro inertness and stability. In parallel, we established a fully quantitative SPECT/CT imaging workflow to characterize the dual-isotope imaging potential of this radiocomplex. This included dedicated phantom experiments to determine isotope specific spatial resolution, uniformity, and calibration factors for ^197m^Hg and ^197g^Hg independently. To address the challenge of overlapping photon emissions and the parent–daughter decay relationship between the two isomers, we implemented Bateman equation–based decay corrections to enable accurate time-resolved quantification of each isomer. To facilitate reproducible and accessible data processing, we developed *HgQuant*—a Python-based tool that automates Bateman decay correction for both imaging and gamma counting workflows.

Using this framework, two preclinical imaging studies were performed to quantitatively image ^197m^Hg and ^197g^Hg: one involving administration of [^197m/g^Hg]Hg-tetrathiol, and a comparative control study employing [^197m/g^Hg]HgCl_2_. Imaging results were validated with ex vivo biodistribution analyses, quantifying the metastable and ground states separately to ensure isotopic correlation. Together, this work demonstrates the feasibility of accurate, isotope-resolved SPECT imaging with the ^197m/g^Hg theranostic pair, enabled by a chemically robust tracer and a rigorous quantitative imaging pipeline. These results establish a foundation for future development of ^197m/g^Hg-based radiotheranostics.

## Methods

### General materials and methods

All solvents and reagents were purchased from commercial suppliers and used as received unless otherwise noted. Ultrapure concentrated hydrochloric acid (HCl, 99.99% trace metal grade, 37%), sodium hydroxide (NaOH, ACS reagent, ≥ 97%, pellets), dimethyl sulfoxide (DMSO), octanol, pentaerythritol tetrakis (3-mercaptopropionate), *L*-glutathione, metal salts (ZnCl_2_, FeCl_3_, CuCl_2_, MgCl_2_ and CoCl_2_) and human serum were purchased from Sigma Aldrich (St. Louis, MO). 20 × PBS Tween-20 buffer was purchased from Thermo Fisher Scientific Inc. (Waltham, MA) and diluted with ultrapure water to 1x. Millipore system (Direct-Q® 3UV with Pump, 18 MΩ cm^−1^) provided ultrapure water. Deuterated solvents used for NMR analysis were purchased from Sigma or Cambridge Isotope Laboratories Inc. (Tewksbury, MA) and exhibited an isotopic purity between 99.9 and 99.8%. All NMR spectra were recorded on a Bruker AVANCE III 600 MHz QCI cryoprobe, Bruker AVANCE III 500 MHz, or Bruker AVANCE III 400 MHz instruments. Chemical shifts are reported in parts per million (ppm) and are referred to the residual solvent peak. Multiplicity is reported as follows: s = singlet, t = triplet, m = multiplet, and br = broad peak. The coupling constants (*J*) are reported in hertz (Hz). High-resolution electrospray-ionization mass spectrometry (ESI-HRMS) was performed on an Agilent 6210 time-of-flight instrument (TOF). The radiolabeling of ligands was monitored using silica-impregnated instant thin-layer chromatography paper (iTLC-SG, Agilent Technologies, Santa Clara, CA, USA). Data were analyzed on an Eckert & Ziegler AR-2000 TLC scanner and processed with Eckert & Ziegler WinScan software (Hopkinton, USA). A Capintec CRC-55tR dose calibrator well counter set at the ^197g^Hg calibration (calibration number: 197) was used to measure activity; Capintec activity readings were cross-referenced with the gamma spectrometer value to ensure accuracy in the reading values. The Capintec was used to measure the activity before radiolabeling reactions. Bio-rad Mini-PROTEAN Tetra Vertical Electrophoresis Cell instrument was used for all Sodium Dodecyl Sulphate—PolyAcrylamide Gel Electrophoresis (SDS-PAGE) measurements with 4–20% Mini-PROTEAN® TGX™ Precast Protein Gels. The SDS-PAGE gel electrophoresis reagents, including the MW standards, TGS buffer, Laemmli sample buffer, and Bio-SafeTM Coomassie stain, were all also purchased from Bio-rad. All work with radionuclides at TRIUMF was undertaken in shielded fume hoods to minimize the dose to experimenters (and special precautions were used to prevent contamination).

***Caution!!!*** [^197m/g^Hg]Hg^2+^ produces ionizing radiation and should be handled in laboratories approved for radioactive work using safe lab practices.

***Caution!!!**** Mercury is a toxic heavy metal, and its compounds should be treated accordingly.*

### Preparation of non-radioactive ^nat^Hg-Tetrathiol complex for NMR studies

The [^nat^Hg]Hg^2+^complexes were prepared via the addition of 50 µL of the pentaerythritol tetrakis(3-mercaptopropionate) ligand solution (10^–2^ M, in DMSO-*d*_*6*_) and 5 µL of HgCl_2_ (10^–1^ M, in DMSO-*d*_*6*_), resulting in an equimolar metal-to-ligand ratio complex. The solutions were then diluted up to 450 µL in DMSO-*d*_*6*_ for NMR experiments at both room temperature and 50 °C. Complex formation was confirmed via ESI-HRMS. A solution of the ligand was prepared using the same procedures as above in order to compare the ligand and the complex. All data were collected using standard Bruker parameters and processed with MestReNova 14.1.2-25024 software.

### [^nat^Hg(Tetrathiol)]^2−^

ESI-HRMS *m*/*z* calcd. for [C_17_H_25_O_8_S_4_Hg]^−^ 687.014; found 687.025 [Hg(HTetrathiol)]^−^ or [Hg(Tetrathiol) + H]^−^. ^1^H NMR (400 MHz, DMSO-d_6_) δ 4.15 (s, 8H), 3.15–3.05 (m, 8H), 2.84–2.76 (m, 8H). ^13^C{^1^H} NMR (151 MHz, DMSO-d_6_) δ 172.37, 60.25, 40.03*, 22.86. *Identified by HSQC correlation; peak under DMSO solvent peak.

### H_4_Tetrathiol

ESI-HRMS *m*/*z* calcd. for [C_17_H_28_O_8_S_4_ + NH_4_] 506.101; found 506.103 [H_4_Tetrathiol + NH_4_]^+^. ^1^H NMR (400 MHz, DMSO) δ 4.14 (s, 6H), 4.07 (s, 2H), 2.72–2.60 (m, 16H), 2.50–2.42 (m, 4H). ^13^C{^1^H} NMR (151 MHz, DMSO) δ 171.47, 171.35, 62.97, 62.58, 38.44, 38.37, 19.66, 19.64.

### Density theory calculations

Density functional theory (DFT) calculations were performed using the Gaussian 16 (Revision B.01) program package with the Becke, 3-parameter, Lee–Yang–Parr (B3LYP) functional (Becke 1993; Frisch et al. 2016). Non-metallic atoms (C, H, O, and S) were modelled using the triple-ζ 6-311G** basis set (Krishnan et al. 1980; McLean and Chandler 1980), while the Stuttgart Dresden (SDD) small-core effective core potential (ECP) with the associated SDD basis set was utilized for Hg^2+^ to account for scalar relativistic effects (as obtained from Basis Set Exchange) (Häussermann et al. 1993; Pritchard et al. 2019; Feller 1996; Schuchardt et al. 2007). Grimme's dispersion correction with Becke-Johnson damping (D3-BJ) was applied to account for empirical dispersion effects (Grimme et al. 2010, 2011). All geometries were optimized in gas phase without imposing any symmetry constraints before solvation modeling. All ligands and complexes were optimized in aqueous solution (dielectric constant ε = 78.36) using the polarizable continuum model with the integral equation formalism variant (IEFPCM), which creates solvent cavities via a set of overlapping spheres (Cossi et al. 2003). Frequency calculations confirmed the absence of imaginary frequencies, indicating that the optimized molecular geometries correspond to true minima on their respective potential energy surfaces. Single-point energy calculations were performed at the same level of theory. Unless specified, all calculations were performed at 25 °C and 1 atm. Initial input geometries were constructed manually and modelled at the MM level using the Avogadro software prior to optimization using DFT. Thermodynamic calculations for Gibbs free energy (Δ*G*) were calculated using Equations S1 and S2 in the supporting information.

### Production and quantification of mercury-197m/g

Production of no-carrier added [^197m/g^Hg]Hg^2+^ was achieved through proton irradiation of natural gold (Au) targets via the ^197^Au(p,n)^197m/g^Hg nuclear reaction at the TR13 (13 MeV) cyclotron at TRIUMF—Canada’s particle accelerator center, following previously published procedures, with calculated rate of productions of 4 MBq/µA·h for ^197m^Hg and 2.9 MBq/µA·h for ^197g^Hg (Chen et al. 2023). Briefly, Au targets were prepared by the addition of 200–270 mg of Au foil to a 10 mm diameter indent (0.25 mm deep) of a tantalum backing (1 mm in thickness) and melted thereon in a furnace at 1250 °C (Rd-G–RD Webb Company–Natick MA, USA). Following proton irradiation (4 h, 30 µA), the Au target was dissolved in *aqua regia* (3 mL), and the solution was then loaded onto a prepared column of LN resin (5 g, 25 mL reservoir). ^197m/g^Hg^2+^ was eluted in 6 M HCl (4 mL) while the ^197^Au was retained on the resin. The ^197m/g^Hg^2+^ solution matrix was then exchanged to a 0.1 M HCl solution by multiple steps of evaporation and reconstitution. The final activity ranged from 90 to 140 MBq of ^197m/g^Hg^2+^ obtained as HgCl_2_ in 250–350 μL 0.1 M HCl. The radionuclide purity was evaluated using gamma (γ)-ray spectroscopy on an N-type co-axial high-purity germanium (HPGe) gamma spectrometer (CANBERRA, Mirion Technologies, Inc., San Ramon, CA, USA), calibrated with a 20 mL ^152^Eu and ^133^Ba source. Samples were prepared by mixing aliquots of ^197m/g^Hg^2+^ activity (1.2 MBq) with deionized water in a 20 mL glass vial to make a 20 mL sample and measured at a distance of 150 mm from the detector for 10 min, ensuring dead times were below 10%. Spectra were analyzed using Genie-2000 software, using the 133.98 keV (*I*_γ_ = 33.5%) and 164.97 keV (*I*_γ_ = 0.2618%) γ-lines of ^197m^Hg, and the 77.35 keV (*I*_γ_ = 18.7%) γ-line of ^197g^Hg for activity calculations (decay radiation referenced from IAEA.org Chart of Nuclides) (Huang and Zhou 2005). The radionuclidic purity was > 99%.

### [^197m/g^Hg]Hg^2+^ radiolabeling studies

The **H**_**4**_**Tetrathiol** ligand was made up as stock solution (10^–3^ M) in DMSO. A serial dilution was used to prepare ligand solutions at 10^−4^, 10^−5^ M, 10^−6^ M, and 10^−7^ M in DMSO. An aliquot (10 μL) of each ligand solution (or DMSO, for negative controls) was diluted with ammonium acetate buffer (1 M; pH 7) such that the final reaction volume was 100 μL after addition of activity. [^197m/g^Hg]HgCl_2_ (1–1.2 MBq, 3–10 μL) was added and mixed gently to begin the radiolabeling reaction at 80 °C, 37 °C, or room temperature. Complex formation was monitored for each reaction by acquiring the non-isolated percentage radiochemical yield (%RCY) at 1 h. This was achieved by extracting an aliquot (10 μL) of the reaction solution and adding it to an equal volume of dimercaptosuccinic acid (DMSA) solution (50 mM, pH 5, 10 μL). The quenched solution was gently mixed and analyzed by spotting a portion (10 μL) of the mixture onto the bottom of an iTLC-SG plate (1 cm × 10 cm, baseline at 1 cm) and then developed using DMSA solution (50 mM, pH 5) as the mobile phase. Under these conditions, the [^197m^Hg]Hg^2+^-complexes remain at the baseline (*R*_f_ = 0), while the unchelated, 'free' ^197m/g^Hg^2+^ migrates towards the solvent front (*R*_f_ = 1). TLC plates were analyzed on an Eckert & Ziegler AR-2000 TLC scanner and processed with Eckert & Ziegler WinScan software. Radiolabeling yields were calculated by integrating the peaks in the radio-chromatogram.

### Stable metal competition assay

To the [^197m/g^Hg][Hg(Tetrathiol)]^2−^ (prepared as described above) or radiolabeling controls (DMSO instead of the ligand) 10 µL of a 10 mM metal cocktail (solution of biologically relevant metals, ZnCl_2_, FeCl_3_, CuCl_2_, MgCl_2_ and CoCl_2_), 10 mM AuCl_3_ or 10 mM of HgCl_2_, in water was added. The final solution contained tenfold excess of stable metal to the chelators and 10^6^-fold excess of the stable metal to ^197m/g^Hg in solution. The mixtures were incubated at RT over 113 h. The proportion of intact radiolabeled complex was monitored over the course of 113 h using iTLC-SG and DMSA solution (50 mM, pH 5) as the mobile phase. Under these conditions, uncomplexed ^197m/g^Hg^2+^ resulting from stable metal transchelation traveled to the solvent front (*R*_f_ = 1) while intact [^197m/g^Hg][Hg(Tetrathiol)]^2−^ remained at the baseline (*R*_f_ = 0).

### L-Glutathione (GSH) competition assay

GSH competition assay procedures closely followed those previously developed by our group (Randhawa et al. 2023). To the [^197m/g^Hg][Hg(Tetrathiol)]^2−^ (prepared as described above) or radiolabeling controls (DMSO instead of the ligand) was added to a 50 mM *L*-glutathione (GSH) solution (1:22 *v*/*v* GSH:reaction solution dilution), and the mixtures were incubated at 37 °C over 72 h. The final GSH concentration was chosen to mimic in vivo conditions within cells (2.12 mM) (Dickinson and Forman 2002). The proportion of intact radiolabeled complex was monitored over the course of 72 h using iTLC-SG and *L*-glutathione (50 mM) as the mobile phase. Under these conditions, uncomplexed ^197m/g^Hg^2+^ resulting from GSH transchelation traveled to the solvent front (*R*_f_ = 1) while intact [^197m/g^Hg][Hg(Tetrathiol)]^2−^ remained at the baseline (*R*_f_ = 0).

### Human serum stability assay

To the [^197m/g^Hg][Hg(Tetrathiol)]^2−^ (prepared as described above) or radiolabeling controls (DMSO instead of the ligand) was diluted in human serum (1:1 *v/v* dilution), and the solutions were incubated at 37 °C over 72 h. The metal-complex stabilities were monitored over 72 h using SDS-PAGE following our previously reported protocols (Randhawa et al. 2023; Tosato et al. 2024). At each time point, an aliquot (10 μL) of the reaction mixture was mixed with Laemmli sample buffer (10 μL) and was directly loaded onto the SDS-PAGE gel. The SDS-PAGE was run at ambient temperature and 150 V until the dye front reached the resolving gel (1 h). Following electrophoresis, the gel was scanned with the radio-TLC scanner to determine the percentage of intact complex. The same protocol was used with free [^197m/g^Hg]Hg^2+^ and the ^197m/g^Hg^2+^-complexes diluted in phosphate-buffered saline (PBS) (5 µL; 1:1 *v/v* dilution) to assess their electrophoretic mobility.

### LogD_7.4_ measurements

Aliquots of [^197m/g^Hg][Hg(Tetrathiol)]^2−^ (prepared as described above) (10 μL) were added to a biphasic mixture of *n*-octanol (700 μL) and phosphate buffered saline (PBS, 700 μL, pH 7.4). The mixture was vortexed for 2 min at ambient temperature and then separated via centrifugation (10 min, 3000 rpm). Aliquots of *n*-octanol (100 μL) and PBS (100 μL) were collected, and the activity in each portion was determined via gamma spectroscopy. LogD_7.4_ is defined as log_10_[(activity in *n*-octanol phase)/(activity in buffer phase)].

### ^197m/g^Hg phantom studies

To characterize the quantitative imaging capabilities of ^197m^Hg and ^197g^Hg, three dedicated phantom experiments were conducted: point source, resolution, and uniformity. These experiments were designed to determine isotope-specific calibration factors, assess spatial resolution and contrast recovery, and evaluate quantification uniformity across large homogeneous regions. The results served to benchmark imaging accuracy, validate isotope separation strategies, and ensure robust quantification ahead of in vivo applications. Table 1 summarizes the key parameters and objectives of each phantom experiment.

**Table 1:** Parameters and purposes for each of the phantom imaging experiments

Phantom	Total activity (MBq)	V (mL)	Activity Concentration (MBq/mL)	Scan time (min)	Purpose
*Point Source*	3.440 (g) 0.819 (m)	0.052	66 (g) 15.7 (m)	10	Isotope-specific calibration factors for quantitative imaging
*Resolution*	9.65 (g) 2.28 (m)	0.637	15.1 (g) 3.57 (m)	30	Spatial resolution, contrast, and accuracy of quantification in small volumes
*Uniformity*	20.8 (g) 4.83 (m)	5.8	3.58 (g) 0.832 (m)	^*a*^	Background uniformity and quantitative accuracy across large homogeneous regions

^a^Scan time for the uniformity phantom was varied across a 120 h imaging schedule to assess decay kinetics and longitudinal consistency (t = 0, 2.0, 3.7, 23.3, 26.3, 31.7, 47.0, 70.1, 95.4, 118.5 h)

### SPECT acquisition and image reconstruction

All phantom experiments were performed using the VECTor SPECT/CT imaging system (MILabs, Utrecht, Netherlands) (Goorden et al. 2013) a multimodal preclinical imaging platform featuring modular cylindrical multi-pinhole collimators optimized for sensitivity and spatial resolution across a range of photon energies. In this study, we investigated the feasibility of in vivo quantitative SPECT imaging of both ^197m^Hg and ^197g^Hg, using the extra ultra-high sensitivity (XUHS) collimator (54 pinholes of 2.0 mm diameter per pinhole, 46 mm tube diameter). This collimator is specifically designed for imaging medium- and low-energy photons, providing exceptional sensitivity even at minimal activity levels (Ivashchenko et al. 2015). 

**Fig. 3.**
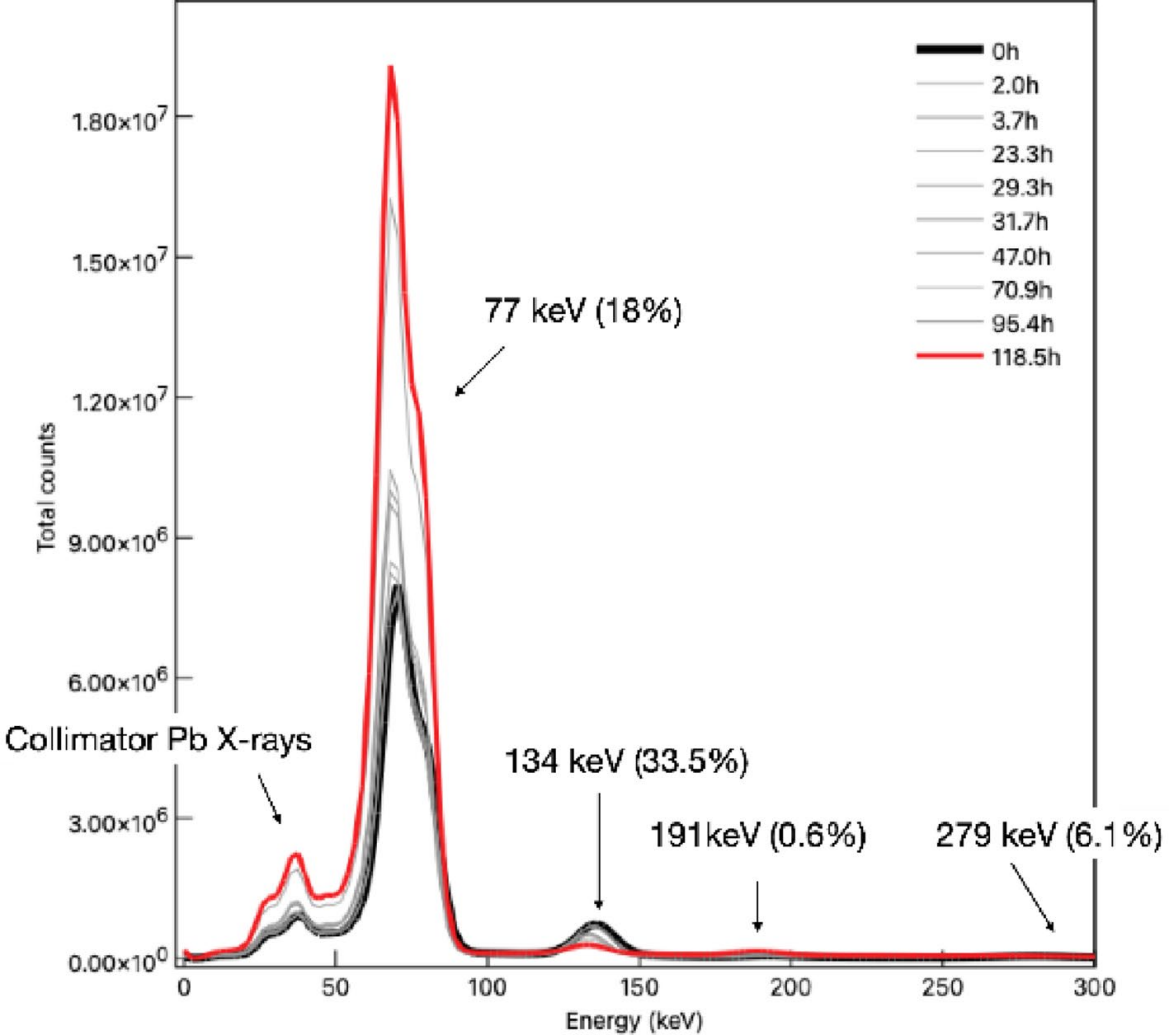
^197m^Hg/^197g^Hg gamma spectrum over time from uniform phantom produced with VECTor/CT imaging system

One of the principal challenges in quantitative imaging of the ^197m^Hg/^197g^Hg pair is their simultaneous presence, stemming from the co-production via ^197^Au(p,n) reaction. The isotopes differ in both half-life and gamma emission spectra. ^197m^Hg has a shorter half-life (23.8 h) and emits gamma photons at 134 and 279 keV, while ^197g^Hg, with its longer half-life (64.1 h), has dominant gamma emission at 77.3 keV (Fig. 1). Furthermore, significant overlap occurs in the 70–80 keV region, where both isotopes contribute low-energy photons and characteristic X-rays, complicating their separation in SPECT images. This coexistence complicates quantitative imaging, requiring optimized energy window selection and scatter correction to accurately separate their contributions. To address this issue, separate energy windows were used to reconstruct images for each radioisotope, ensuring proper differentiation. Therefore, ^197g^Hg images were reconstructed using the photopeak window centered at 77 keV, applying a 40% width photopeak energy window and 6% width upper and lower scatter windows. ^197m^Hg images were reconstructed using the 134 keV gamma emission, with 15% width photopeak energy window and 4% width upper and lower scatter windows (Fig. 3).

All images were reconstructed using the pixel ordered subsets expectation maximization algorithm (POSEM) (Branderhorst et al. 2010) with 16 subsets and 6 iterations and an isotropic 0.4 mm voxel grid. The reconstructed SPECT images were then registered to the CT images, resampled to an isotropic voxel size of 0.16 × 0.16 × 0.16 mm^3^ voxels, and attenuation correction was performed using the CT-based non-uniform Chang method (Wu et al. 2010). Image reconstruction, registration, and attenuation correction were all conducted using the manufacturer’s software. Reconstructed images were analyzed using AMIDE v.1.0.5 and custom Python (v3.8.8, https://python.org) scripts. The NiBabel package (v3.2.1, https://nipy.org/nibabel) was used to read SPECT and CT NIfTI files, while visualization was performed with Matplotlib (v3.5.1, https://matplotlib.org). The scikit-image library (v0.18.1, https://scikit-image.org) facilitated the definition of regions of interest (ROIs) and line profiles, and NumPy (v1.22.3, http://numpy.scipy.org) was used for data analysis.

### Activity quantification and decay correction

To determine the activity of ^197m^Hg and ^197g^Hg, aliquots of a ^197g/m^Hg stock solution were collected and analyzed using gamma spectroscopy. Since ^197m^Hg has a much shorter half-life than ^197g^Hg, equilibrium cannot be assumed, and their activities must be calculated independently, rather than assuming a constant ratio between them. To account for this time dependent decay behaviour, the Bateman equations were applied to describe the activity of each isotope over time. Activity decay corrections were applied to align measurements with the exact time of imaging using the following equations:

1 $${A}_{Hg-197m}(t)={A}_{Hg-197m}(0)\cdot {e}^{-{\lambda }_{m}t}$$

2 $${A}_{Hg-197g}(t)=\frac{{\lambda }_{IT\left(m\right)}}{{\lambda }_{g}-{\lambda }_{IT\left(m\right)}}{\left(\frac{{\lambda }_{g}}{{\lambda }_{m}}\right)A}_{Hg-197m}(0)\left({e}^{-{\lambda }_{IT\left(m\right)}t}-{e}^{{-{\lambda }_{g}t}}\right)+{A}_{Hg-197g}(0){e}^{-{\lambda }_{g}t}$$

where ${A}_{Hg-197m}(0)$ and ${A}_{Hg-197g}(0)$ are the initial ^197m^Hg and ^197g^Hg activity measured independently via gamma spectroscopy, ${\lambda }_{g}$ and ${\lambda }_{m}$ are the physical decay constants for ^197g^Hg and ^197m^Hg, respectively, while ${\lambda }_{IT\left(m\right)}$ is the partial decay constant of ^197m^Hg, which takes into account grow-in of ^197g^Hg from the 91.4% branching decay via isomeric transition (IT) of ^197m^Hg (${\lambda }_{IT\left(m\right)}=0.914\times {\lambda }_{m})$.

To streamline and standardize this correction process, we developed *HgQuant*, a custom Python-based software tool that automates activity calculation for the ^197m^Hg/^197g^Hg pair (Fig. S13). *HgQuant* allows researchers to calculate and visualize time-resolved activity distributions based on initial activity, decay constants, and user-defined timepoints of imaging or biodistribution sampling.

*HgQuant* was employed throughout this study for all phantom and biological experiments, ensuring reproducible, isotope-specific quantification and enabling accurate comparisons across datasets. The tool is available upon request or via GitHub: https://github.com/cristinarod2/HgQuant.

#### Point source phantom

The counts in the reconstructed images were converted into units of activity concentration by applying isotope-specific experimental calibration factors for ^197m^Hg and ^197g^Hg, following a procedure previously described by some of the authors elsewhere (Esquinas et al. 2018; Koniar et al. 2022). Briefly, a uniform ^197m/g^Hg point source in an Eppendorf tube containing 66.0 MBq/mL of ^197g^Hg and 15.7 MBq/mL of ^197m^Hg in 0.1 M HCl was scanned for 10 min in a single bed position. The acquired data were reconstructed using the same parameters and corrections as those described above. To establish the calibration factors, a volume of interest (VOI) was defined in the SPECT image of the point source to correlate the average voxel intensity with the activity concentration determined via HPGe gamma spectroscopy for each isotope. The resulting calibration factors were applied across all SPECT datasets, including the contrast, uniformity, and resolution phantoms, as well as in vivo scans, ensuring consistent and quantitative assessment of radiotracer uptake throughout the study.

#### Resolution phantom

The resolution phantom was filled with a solution containing 15.1 MBq/mL of ^197^Hg and 3.57 MBq/mL of ^197m^Hg. SPECT images of the resolution phantom were analyzed as described in previously reported work by Grimme et al. (2011). Briefly here, interrod contrast is a metric for the apparent activity within the rods and between the rods and determines the minimum resolvability for the imaging system. Interrod contrast > 20% is defined as resolvable. Contrast to noise ratio (CNR) is an image quality metric that evaluates signal detectability by normalizing the interrod contrast to the variability of activity measurements among rods with the same diameter. Higher CNR values indicate better differentiation of rods from background noise. Lastly, the recovery coefficient (RC) is a metric for the quantitative accuracy of SPECT images in small hot volumes by determining the ratio of the apparent activity in the rod to the known activity determined by gamma spectroscopy.

#### Uniformity phantom

At time of first image, the uniformity syringe contained 3.58 MBq/mL of ^197g^Hg and 0.832 MBq/mL of ^197m^Hg. The uniformity phantom was imaged (*n* = 10) over the course of 120 h (5 d) to measure the decay of the metastable and ground state isomers and the accuracy of quantitative image-derived activity measurements compared to the known activities inside the syringe. For each acquisition, the resultant image was analyzed for its apparent activity concentrations of ^197^Hg and ^197m^Hg. A circular region of interest (ROI) with a 6 mm radius was placed in 5 non-consecutive transaxial slices within the uniform activity region. The mean and standard deviation of activity were recorded for each isotope. The measured activity concentrations over time were fitted with mono-exponential decay models to characterize the decay kinetics of both isotopes. These decay curves were then compared to the known physical half-lives to evaluate imaging accuracy and consistency over time.

### Tracer preparation for in vivo SPECT imaging and ex vivo biodistribution.

For animal injection, the tracer was prepared at high molar activity. Radiolabeling of **H**_**4**_**Tetrathiol** (10^–2^ M in DMSO; 40 μL for imaging, 20 μL for bioD) (or DMSO, for [^197m/g^Hg]HgCl_2_ mice) with [^197m/g^Hg]HgCl_2_ (imaging: 26.7 MBq total m + g, 62 μL; bioD: 13.04 MBq total m + g, 31 μL) was performed in a mixture of ammonium acetate buffer (1 M; pH 7; 198 μL for imaging or 149 μL for bioD) and 6 M NaOH (72 μL or 38 μL required to achieve a pH of 7.5 after addition of activity, for imaging or BioD labeling, respectively). The reaction was allowed to stand for 1 h at 37 °C and quantitative (> 99%) non-isolated percentage radiochemical yield (%RCY) was confirmed via the iTLC methods mentioned above. Further, 100 µL of the reaction was diluted with 50 µL of PBS Tween-20 (0.01 M sodium phosphate, 0.15 M NaCl, 0.05% Tween-20) for imaging mice, or 50 µL of the reaction was diluted with 100 µL of PBS Tween-20 for BioD mice. The tracers were prepared immediately before injection to avoid radiolysis and minimize the tracer loss to any surfaces due to its lipophilicity. The injected doses of each isomer, ^197m^Hg and ^197g^Hg, were quantified by measuring an aliquot of known volume of each tracer preparation via HPGe gamma spectroscopy and decay corrected for time of injection using the Eqs. (1) and (2).

### In vivo imaging

The study was conducted in compliance with the guidelines set by the Canadian Council on Animal Care (CCAC) and approved by the Animal Care Committee (ACC) at the University of British Columbia (A20-0132). A total of eight healthy male C57BL/6 mice weighing approximately 30 g for group 1 and 25 g for group 2 were used in the study. The mice were divided into two experimental groups: group 1 (*n* = 4) received free [^197m/g^Hg]HgCl_2_, while group 2 (*n* = 4) received [^197m/g^Hg]Hg-Tetrathiol. Within each group, one mouse was allocated for imaging, while the rest were designated for ex vivo biodistribution analysis. For SPECT imaging, the designated mice were anesthetized using isoflurane via a precision vaporizer. Induction was achieved with a mixture of 5% isoflurane in oxygen, followed by maintenance with 1.5–2.5% isoflurane in oxygen. Once anesthetized, each imaging mouse received an intravenous (IV) injection via the lateral tail vein of 150 μL of [^197m/g^Hg]HgCl_2_ (0.492 MBq ^197m^Hg + 3.171 MBq ^197g^Hg) or [^197m/g^Hg]Hg-Tetrathiol (0.899 MBq ^197m^Hg + 4.230 MBq ^197g^Hg) in PBS Tween-20 (0.01 M Na Phosphate, 0.15 M NaCl, 0.05% Tween-20).

The animals were immediately placed in the scanner and whole-body scans were acquired dynamically during 120 min post-injection (6 frames × 20 min/frame). Throughout the entire scanning procedure, mice were kept under isoflurane anesthesia and body temperature was maintained constant using a heated imaging bed. Consistent with the phantom studies, the scanner was equipped with an extra-ultra high-sensitivity collimator (XUHS) and followed by a cone-beam CT scan (55 kV, 615 μA) for anatomical reference and attenuation correction. The animal projection data were reconstructed using the ^197g^Hg (energy window settings: 77 keV, 40% energy window width) and ^197m^Hg (energy window settings: 134 keV, 15% energy window width). These energy window settings yielded the optimum, and most robust quantification results based on the phantom experiments (vide supra). At the end of the imaging study, mice were euthanized via an overdose of isoflurane and treated as the biodistribution animals below.

### Ex vivo biodistribution

Mice received an IV injection of [^197m/g^Hg]HgCl_2_ (0.275 MBq ^197m^Hg + 1.758 MBq ^197g^Hg, 150 μL) or [^197m/g^Hg]Hg-Tetrathiol (0.384 MBq ^197m^Hg + 1.733 MBq ^197g^Hg, 150 μL) (*n* = 3 per group) via the lateral tail vein using a Tailveiner restrainer. After the injection, the mice were returned to their experimental cages and allowed unrestricted movement. Biodistribution studies were conducted 2 h after administration. Mice were humanely euthanized with an overdose of isoflurane followed by cardiac puncture to collect blood pool activity. The organs of interest were fully extracted, cleaned of blood without rinsing, weighed, and analysed for radioactivity using a calibrated gamma counter with the 55–93 keV energy window for ^197g^Hg and the 110–160 keV energy window for ^197m^Hg (Packard Cobra II, Perkin Elmer, Waltham, MA, USA). The stomach and intestines were carefully cleaned prior to analysis to remove residual contents. For tissues and fluids that could not be fully removed (muscle, blood and bone) activity values were extrapolated based on standardised organ weights for age- and sex-matched mice tables to ensure accurate normalization (Davies and Morris 1993; Foster et al. 2014). The stomach and whole intestines were cleaned before analysis. The radioactivity measurements were decay corrected for time of injection using the Eqs. (1) and (2). The results are reported as the percentage of the injected activity per gram of tissue (%IA/g) and percentage of the injected activity per tissue (%IA/organ).

## Results

### Non-radioactive metal complexation with the tetrathiol chelator

**Fig. 4:**
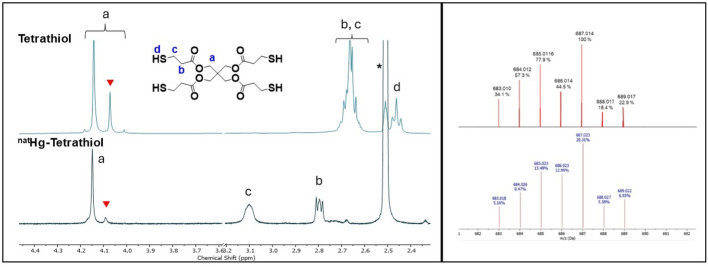
(left) ^1^H NMR spectra of the H_4_Tetrathiol ligand, and its Hg^2+^ complex (600 MHz, DMSO-*d*_6_*, 25 °C). Red triangles: suspected disulfide ‘dimer’ species. (right) ESI^−^ High-resolution mass spectrum (HRMS) of predicted (top) for [C_17_H_25_O_8_S_4_Hg]^−^ and experimentally obtained (bottom) for [Hg(HTetrathiol)]^−^

Prior to radiolabeling with ^197m/g^Hg, the coordination chemistry of ^nat^Hg^2+^ with the tetrathiol ligand was investigated using NMR spectroscopy, high resolution mass spectrometry (HRMS), and density functional theory (DFT) calculations to aid in structural interpretation. For solution studies, complexes were formed by mixing equimolar ligand and HgCl_2_ in DMSO, producing 1:1 metal-to-ligand species characterized by NMR (Figs. 4, S7–S9) and HRMS (Figs. 4, S10–S11).

NMR spectra revealed distinct changes upon complexation. In the free ligand, additional resonances indicated disulfide-linked dimers (Figs. S1–S6); however, these signals diminished upon Hg^2⁺^ addition, suggesting mercury insertion into disulfide bridges (Marston and Wright 1984) and formation of a singular complex. Downfield shifts in proton resonances throughout the complex supported coordination, particularly for protons alpha to the thiol (2.66 → 3.10 ppm) and carbonyl groups (2.66 → 2.80 ppm), consistent with proximity to the electron-deficient Hg^2⁺^ center. Minor shifts near the ester group (4.14 → 4.15 ppm) were also observed. The disappearance of SH resonances confirmed deprotonation and coordination of all four thiol groups. HRMS data showed a signal at *m/z* 687.025 (assigned as [(HTetrathiol)^3−^ + Hg^2+^]^−^), a dominant ion peak at *m/z* 723.000 (assigned as [(H_2_Tetrathiol)^2−^ + Hg^2+^  + Cl^−^]^−^) and fragment at *m/z* 635.001 with the expected mercury isotope distribution (Figs. S10–S11), confirming the formation of a 1:1 metal–ligand complex. Other ion peaks present at higher *m/z* exhibit isotopic distributions consistent with multinuclear mercury-containing species. In particular, the signal at *m/z* 920.955 matches the expected isotope pattern for a binuclear Hg-complex (putatively assigned as [(Tetrathiol)^4−^ + 2Hg^2+^  + Cl^−^]^−^), which would correspond to a 2:1 metal–ligand stoichiometry (Fig. S11). Because electrospray ionization can produce adducts, multimers and in-source association products, MS evidence alone is insufficient to definitely assign molecular formulae. Accordingly, signals at higher *m/z* may be attributed to non-covalent multimers, chloride/solvent adducts, or gas-phase aggregation formed during ionization.

**Fig. 5:**
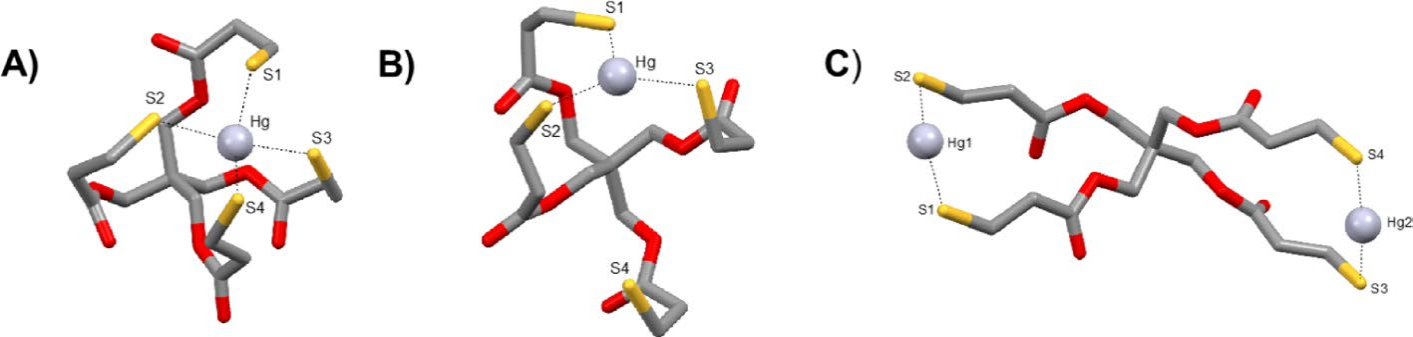
Lowest energy optimized geometries of **A** 4-coordiante Hg-Tetrathiol complex, **B** 3-coordinate Hg-Tetrathiol complex and **C** Hg_2_-Tetrathiol complex modelled at the B3LYP-D3/6-311G**/SDD(Hg)IEFPCM(H_2_O) level of theory. Carbon-bonded hydrogen atoms have been omitted for clarity

The coordination number of Hg^2⁺^ with multidentate acyclic ligands can range from 2 to > 4. To evaluate the preferred coordination mode with Tetrathiol, we performed DFT calculations (B3LYP-D3/6-311G**/SDD(Hg)/IEFPCM(H₂O)) on three potential complexes: a dianionic 4-coordinate species (HgL_1_), a 3-coordinate species (HgL_2_), and a neutral homodimetallic complex (Hg_2_L). All structures optimized to energy minima (Fig. 5), with no evidence of ester carbonyl participation in Hg^2⁺^ binding. Key structural and thermodynamic parameters are summarized in Tables S1–S10.

Gibbs free energy (Δ*G*) calculations showed all complexes to be thermodynamically favorable, with the 4-coordinate complex being the most stable (Δ*G* = –232.38 kcal/mol), followed by the dimetallic (–90.04 kcal/mol) and 3-coordinate (–51.98 kcal/mol) forms (Fig. S12). In HgL_1_, Hg^2⁺^ is bound by four thiolates in a distorted tetrahedral geometry (avg. Hg–S = 2.459 Å), with trans S–Hg–S angles of 124.0° and 146.1°, consistent with NMR observations. The bond lengths range from 2.353 to 2.591 Å, indicating slight asymmetry. A small HOMO–LUMO gap (4.254 eV) further supports the structural stability of the 4-coordinate complex.

### [^197m/g^Hg]Hg^2+^-tetrathiol radiolabeling studies

**Fig. 6:**
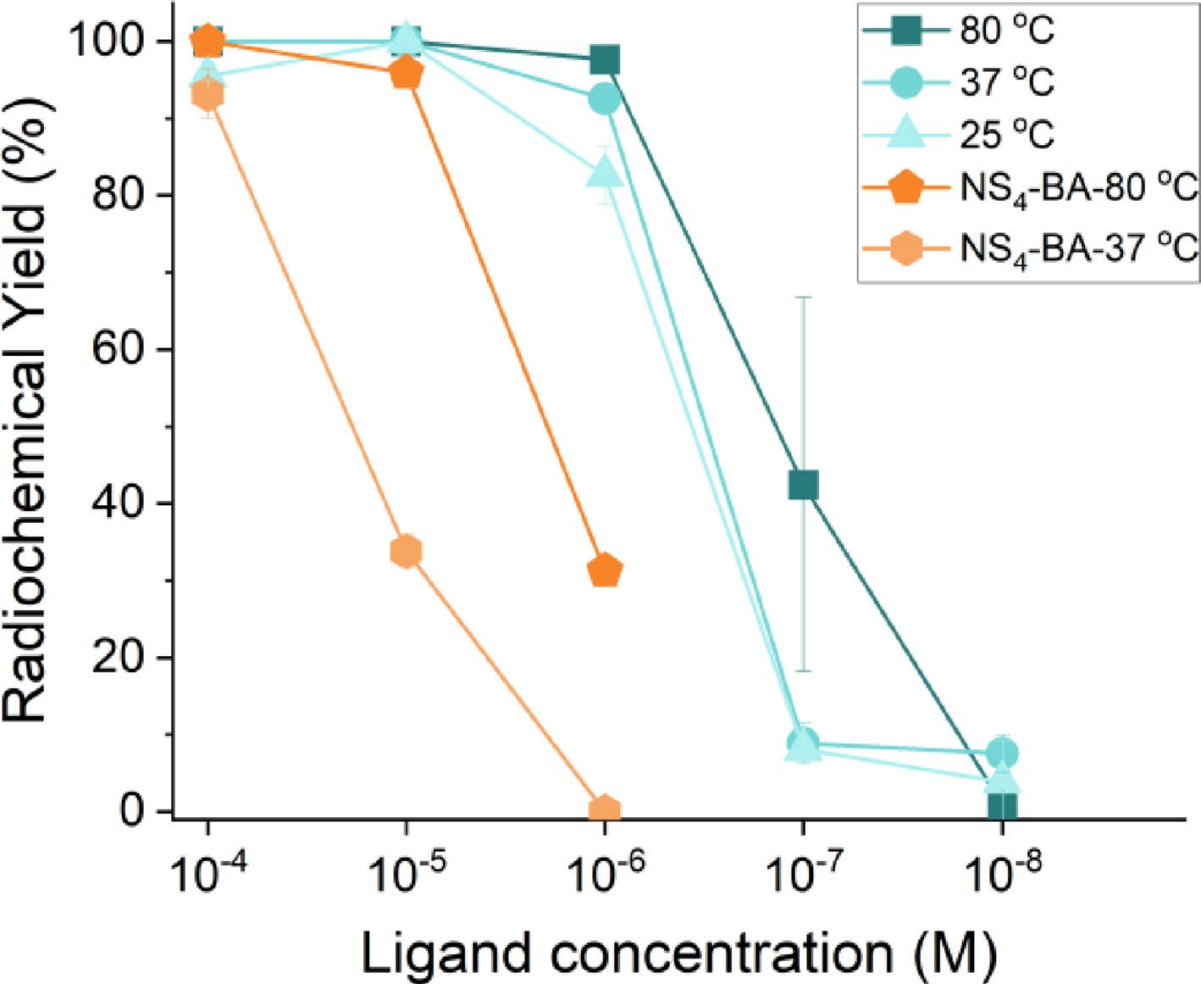
[^197m/g^Hg]HgCl_2_ (~ 1 MBq) radiochemical yields (RCYs) at various ligand concentrations for **Tetrathiol** (1 M NH_4_OAc, pH 7), at 80 °C, 37 °C and 25 °C (1 h reaction time, 100 µL reaction, *n* = 3). [^197m/g^Hg][Hg(NS_4_-BA)]^2+^ 80 °C and 37 °C labeling data is reproduced from Randhawa et al. (2023)

[^197m/g^Hg]Hg^2+^ radiolabeling studies were undertaken with radio-mercury produced on a medical cyclotron (Oram et al. 1996). Ligand concentration-dependent radiolabeling experiments with the sulfur-rich Tetrathiol chelator were conducted at 80 °C, 37 °C and 25 °C (1 h incubation time, at pH 7 in 1 M NH_4_OAc) and compared to the previously reported NS_4_-BA chelator “gold standard” defined in our previous work (Aghevlian et al. 2017). Results are summarized in Fig. 6. At all reaction temperatures, quantitative incorporation yields (> 95%) at 10^–4^ M and 10^–5^ M ligand were achieved. At 10^–6^ M high radiochemical yields (RCYs) of 98 ± 2%, 93 ± 1% and 83 ± 4% were still obtained at temperatures 80 °C, 37 °C and 25 °C, respectively. However, a drastic decrease in the radiochemical yield occurred at a final ligand concentration of 10^–7^ M, yielding 42 ± 27%, 9 ± 3% and 8 ± 5%, at temperatures 80 °C, 37 °C and 25 °C, respectively. At the final ligand concentration tested, 10^–8^ M, RCYs were 1 ± 1%, 8 ± 3% and 4 ± 5% at temperatures 80 °C, 37 °C and 25 °C, respectively. Comparing the RCY of the Tetrathiol to that of the NS_4_-BA, significant improvement in radiolabeling can be observed. At both 80 °C and 37 °C, quantitative labeling of Tetrathiol can be achieved with onefold and tenfold lower ligand concentration, respectively, compared to the NS_4_-BA. The metal:ligand (M:L) ratio achieved at 10^–6^ M for the Tetrathiol labeling at 80 °C is 1:264 (when considering 50% of activity in the metastable state and 50% in the ground state). To-date this is the lowest M:L ratio that has been achieved with [^197m/g^Hg]Hg^2+^ radiolabeling to our knowledge; however, it is close to the ratio (1:671) reported by Gilpin et al. (2021).

### Kinetic inertness of radiolabeled complexes

**Fig. 7:**
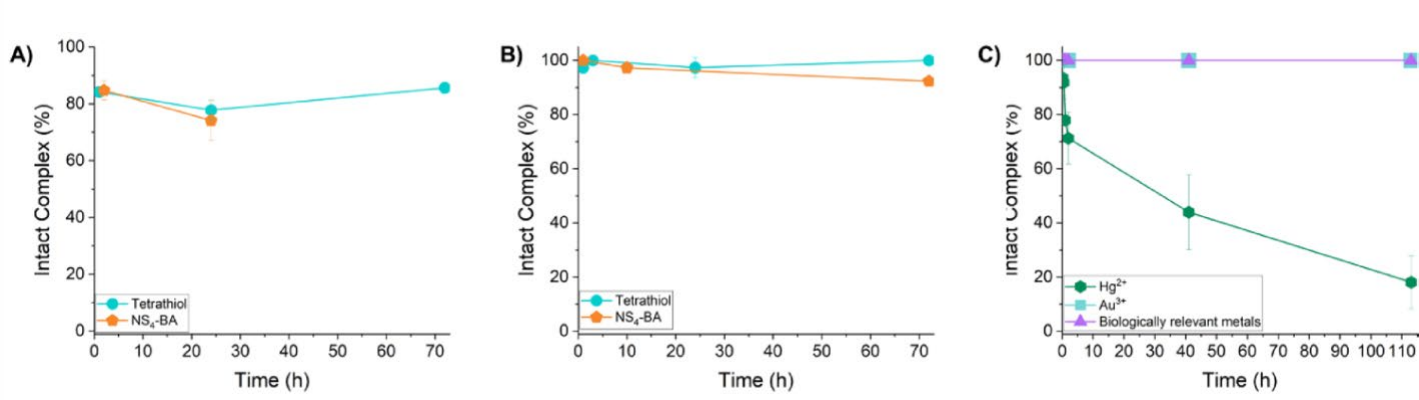
Kinetic inertness of [^197m/g^Hg]Hg-Tetrathiol against **A** Human serum, **B** *L*-Glutathione with comparison to the formally reported NS_4_-BA and **C** Stable AuCl_3_, HgCl_2_ and biologically relevant metals (ZnCl_2_, FeCl_3_, CuCl_2_, MgCl_2_ and CoCl_2_) at 37 °C (*n* = 3) over time. [^197m/g^Hg][Hg(NS_4_-BA)]^2+^ stability data is reproduced from Randhawa et al. (2023).

Following successful quantitative incorporation of [^197m/g^Hg]Hg^2+^ with the tetrathiol chelator, kinetic inertness of the resulting Hg-complex was investigated through a series of in vitro assays against competitors such as human serum, *L-*glutathione (GSH) and biologically relevant metals (ZnCl_2_, FeCl_3_, CuCl_2_, MgCl_2_ and CoCl_2_) as well as excess AuCl_3_ (the target material used in ^197m/g^Hg production) and HgCl_2_ (Fig. 7). In human serum at 37 °C over 72 h, [^197m/g^Hg]Hg-Tetrathiol remained 86 ± 1% intact. In comparison, the NS_4_-BA complex was observed to be 74 ± 7% intact over 24 h, suggesting an increase in inertness with the [^197m/g^Hg]Hg-Tetrathiol. When challenged against GSH, the most abundant thiol-containing small molecule in mammalian cells known to exhibit a high affinity to Hg^2+^, the Hg-tetrathiol complex gave no evidence of decomposition or transchelation, whereas the NS_4_-BA complex demonstrated some lability with 92 ± 1% intact complex over 72 h.

When challenged against a mixture of biologically relevant metals (ZnCl_2_, FeCl_3_, CuCl_2_, MgCl_2_ and CoCl_2_), or AuCl_3_ (tenfold excess compared to chelator and 10^6^-fold excess compared to radiometal), the tetrathiol-complex showed no signs of demetallation over the course of the study (113 h) at ambient temperature (Fig. 7). However, in competition with HgCl_2_, the complex exhibits rapid metal–ligand exchange within 2 h, resulting in 71 ± 10% intact complex, further dissociating to 18 ± 10% intact over 113 h.

### logD_7.4_ measurements of [^197m/g^Hg]Hg-tetrathiol

The hydrophilicity of the radiolabeled complex was evaluated by measuring the partition coefficient between n-octanol and phosphate-buffered saline (PBS, 0.01 M, pH 7.4), utilizing the "shake-flask" method. The radiolabeled complex [^197m/g^Hg]Hg-Tetrathiol was found to have a logD_7.4_ value of 0.35 ± 0.1, indicating the tracer is lipophilic.

### Phantom studies

#### Resolution phantom

**Fig. 8:**
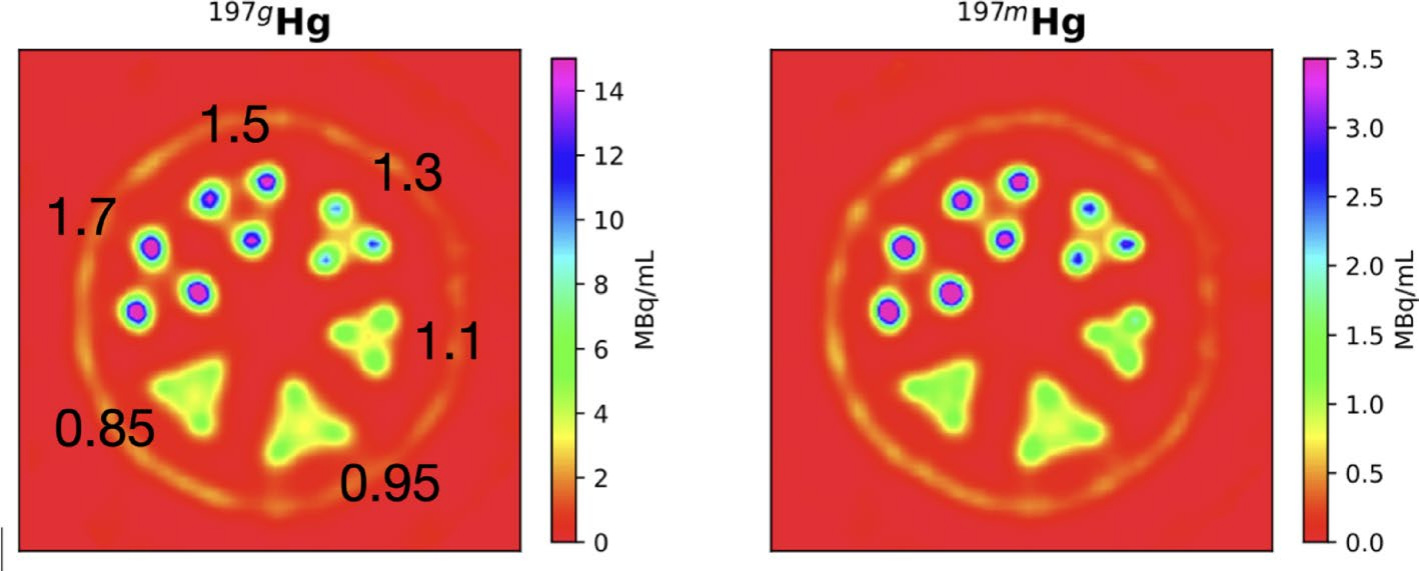
SPECT images of the resolution phantom reconstructed from ^197g^Hg (77 keV) (*left*) and ^197m^Hg (134 keV) (*right*)

**Fig. 9:**
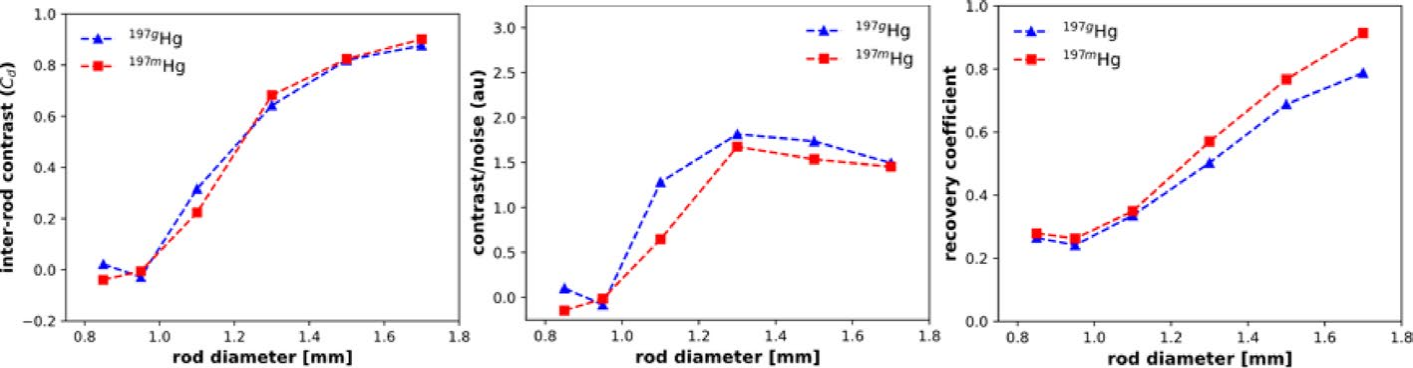
Inter-rod contrast, contrast to noise, and recovery coefficient image quality metrics

A micro-Jaszczak phantom was used to evaluate the spatial resolution and quantitative imaging performance of both ^197m^Hg and ^197g^Hg using the XUHS collimator. As shown in Fig. 8, rods ≥ 1.1 mm in diameter were clearly resolved in images reconstructed from both isotopes, with **inter-rod contrast values exceeding 20%**, the conventional threshold for spatial separability in high-resolution SPECT (Koniar et al. 2024). Quantitative image quality metrics (Fig. 9), including inter-rod contrast, contrast-to-noise ratio (CNR), and recovery coefficients (RCs), highlighted superior performance trade-offs. For the larger rods ^197^^g^Hg demonstrated higher inter-rod contrast and CNR, indicating greater visual detectability and better contrast discrimination in noise-limited conditions. This explains the superior visual appearance of smaller rods in the ^197g^Hg image (Fig. 8).

In contrast, ^197m^Hg showed consistently higher recovery coefficients than ^197g^Hg, especially in larger-diameter rods, suggesting more accurate quantification of true activity concentrations. This is likely due to reduced photon scatter at its higher emission energy (134 keV). While ^197g^Hg performed better in image contrast and noise suppression, ^197m^Hg offered improved fidelity for quantitative analysis. These findings underscore the importance of balancing visual performance and quantitative accuracy when selecting an isotope. While ^197g^Hg may be preferable for high-contrast feature visualization, ^197m^Hg offers advantages in accurate tracer quantification—especially relevant for applications requiring precise kinetic modeling or absolute uptake estimates.

#### Uniformity phantom

**Fig. 10:**
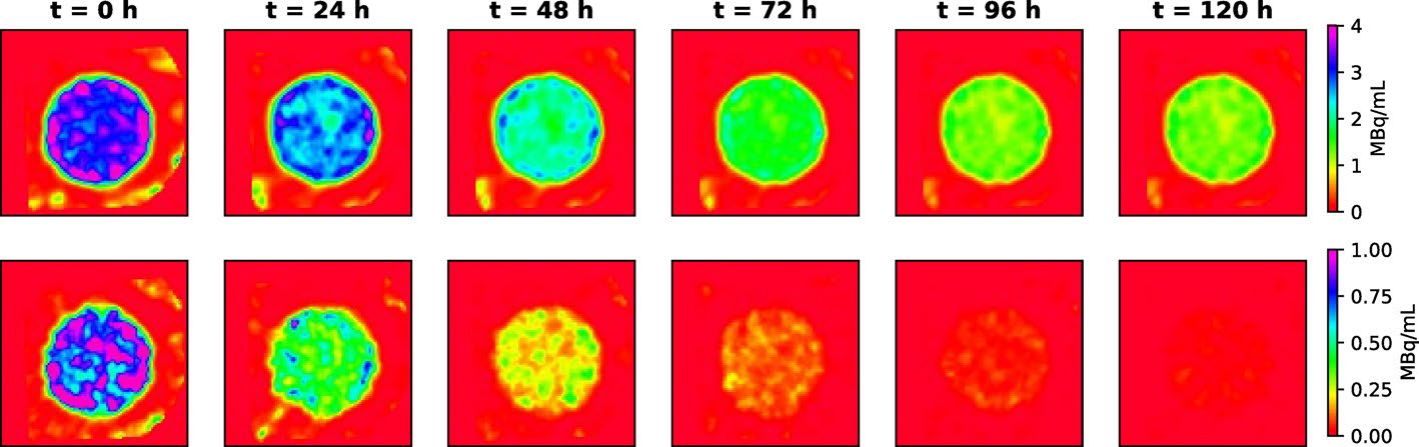
SPECT images of the syringe uniformity phantom acquired over time and reconstructed from ^197g^Hg (*top*) and ^197m^Hg (*bottom*) photon emissions

The uniformity phantom was used to assess the quantitative stability and temporal accuracy of SPECT imaging for ^197m^Hg and ^197g^Hg over extended time points. A 10 mL syringe filled with a uniform distribution of activity was scanned repeatedly over 120 h, and reconstructed images were used to extract apparent activity concentrations in a large central volume of interest (VOI). As shown in Fig. 10, both isotopes produced consistent signal distributions over time, although the ^197m^Hg images (bottom row) exhibited visibly higher noise at later time points due to the shorter half-life and lower residual activity. This aligns with expectations based on the 23.8 h half-life of ^197m^Hg, which decays rapidly compared to the 64.1 h half-life of ^197g^Hg.

**Fig. 11:**
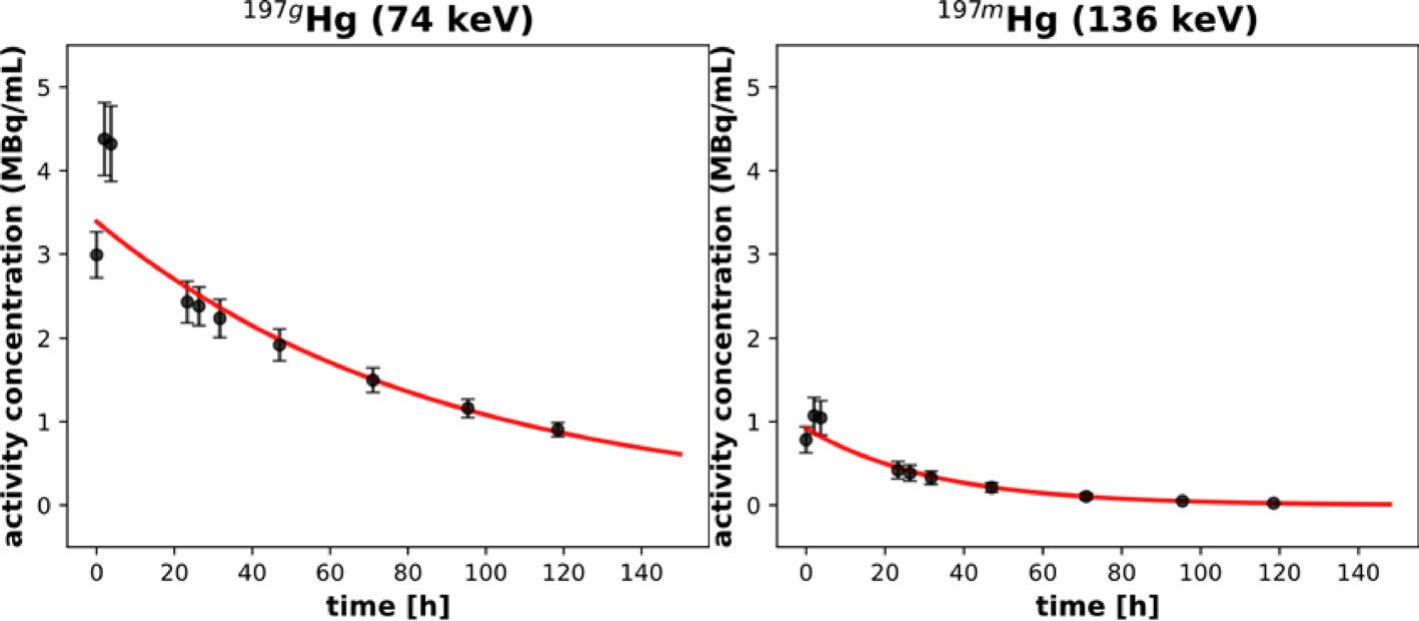
Apparent activity concentration in the uniformity syringe of ^197g^Hg (*left*) and ^197m^Hg (*right*) over time. Data was fit with mono exponential decay model in red. Error bars represent 1 SD

Quantitative analysis of activity concentration decay is shown in Fig. 11. Fitting the time-series data with a mono-exponential decay model yielded effective half-lives of 60.7 ± 5.9 h for ^197g^Hg and 22.4 ± 1.1 h for ^197m^Hg. These values are in excellent agreement with the respective physical half-lives of each isotope (64.1 h and 23.8 h), demonstrating the consistency of quantitative measurements over time. The agreement also validates the accuracy of the attenuation and scatter corrections used in the reconstruction protocols.

This phantom study validates that both isotopes can be reliably quantified over time in uniform volumes. However, the faster signal degradation and increasing image noise observed for ^197m^Hg at later time points suggest practical limitations for longitudinal studies beyond 24–36 h. In contrast, ^197g^Hg offers a more stable imaging window, making it better suited for delayed imaging protocols such as clearance or biodistribution studies.

### In vivo SPECT/ CT and ex vivo biodistribution studies

Given the excellent in vitro inertness of the [^197m/g^Hg]Hg-Tetrathiol, we evaluated the in vivo biodistribution of this tracer in healthy mice and compared its pharmacodynamic profile with that of unchelated [^197m/g^Hg]HgCl_2_ over 2 h. Biodistribution studies were performed after acquisition of the final SPECT/CT scans at 2 h post injection (p.i.) of [^197m/g^Hg]Hg-Tetrathiol and [^197m/g^Hg]HgCl_2_. With the data obtained from phantom studies and gamma counter calibrations, the metastable and ground states were independently quantified in vivo (via SPECT imaging) and ex vivo (via biodistribution studies). Complete biodistribution results are provided in the Supporting Information, Tables S11−S14. Qualitatively, [^197m/g^Hg]HgCl_2_ shows a strong affinity for the kidneys, which is in line with previous studies (Gilpin et al. 2021), while [^197m/g^Hg]Hg-Tetrathiol predominantly accumulates in the spleen and liver, as shown in Fig. 13.

*Ex vivo biodistribution*: As an initial step in our data analysis, we aimed to assess the equivalence in tissue uptake between ^197m^Hg and ^197g^Hg using gamma counter-based biodistribution measurements. Since both are nuclear isomers of the same element, their pharmacodynamic behaviour is expected to be nearly identical.

**Fig. 12:**
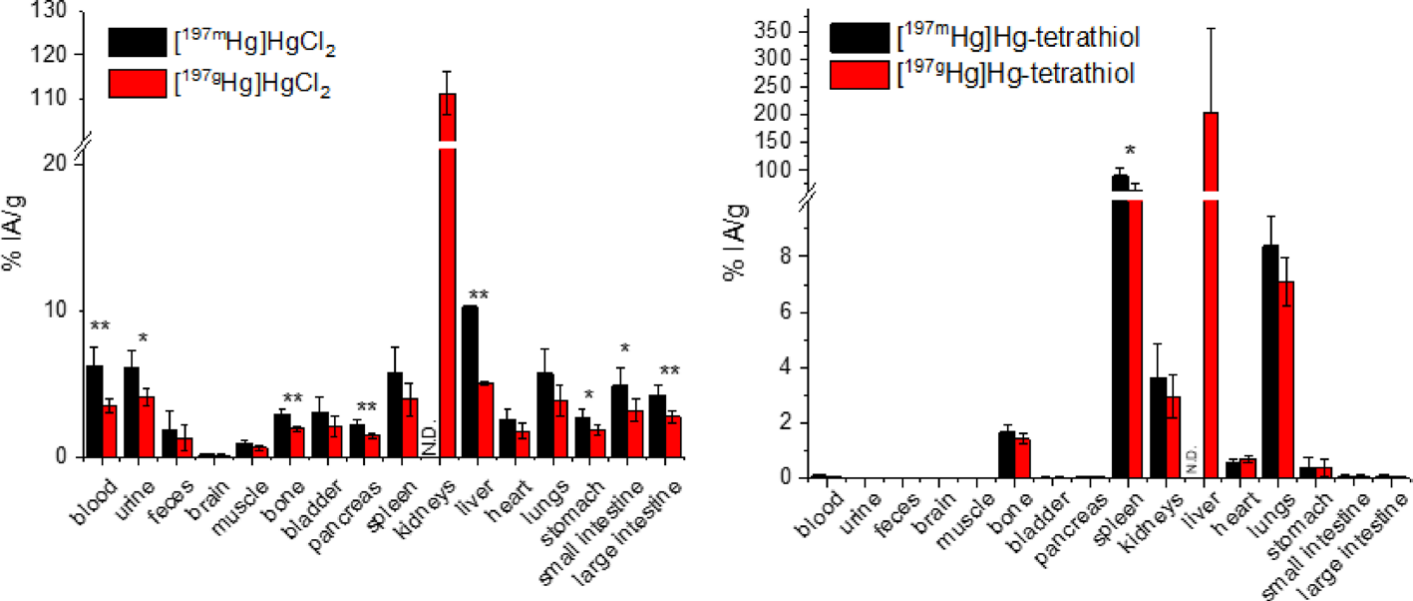
Biodistribution for [^197m^Hg/^197g^Hg]HgCl_2_ (left) and [^197m^Hg/^197g^Hg]Hg-tetrathiol (right) at 2 h post administration in healthy male C57BL/6 mice (*n* = 4 each tracer) represented as percentage of injected activity per gram of tissue (%IA/g); organs measured via gamma counter and quantified for metastable (^197m^Hg; 110–160 keV gamma window) and ground (^197g^Hg; 55–93 keV gamma window) state isomers. Administered activity quantified by HPGe gamma spectroscopy. N.D. = not determined. Student unpaired *t*-test: *p* < 0.05 = *; *p* < 0.01 = **; *p* < 0.001 = ***

However, when comparing the biodistribution of [^197m/g^Hg]HgCl_2_ for each isomer (Table S11, Fig. 12), we consistently observed higher activity for the metastable state. Statistically significant differences in uptake were noted for ^197m^Hg compared to ^197g^Hg in the blood (*p* < 0.01), urine (*p* < 0.05), bone (*p* < 0.01), pancreas (*p* < 0.01), liver (*p* < 0.01), stomach (*p* < 0.05), small intestine (*p* < 0.05), and large intestine (*p* < 0.01). In contrast, for the [^197m/g^Hg]Hg-Tetrathiol tracer (Table S13, Fig. 12), the biodistribution profiles of the two isomers were more closely aligned. Still, a statistically higher uptake of ^197m^Hg was detected in the spleen (*p* < 0.05), one of the major organs of tracer accumulation.

Although true biological differences between the isomers cannot be completely ruled out, the observed discrepancies likely result from overestimation of ^197m^Hg. This was especially evident in organs with high tracer uptake, such as the liver for Hg-tetrathiol and the kidneys for HgCl_2_, where samples left to decay for approximately one month and then recounted yielded decay-corrected ^197m^Hg activities that greatly exceeded the total injected activity. These findings suggest that quantification of ^197m^Hg in such tissues may be affected by significant artifacts, limiting its reliability.

While ^197m^Hg and ^197g^Hg are produced in roughly equal proportions at end of bombardment, decay prior to formulation and injection resulted in a predominance of the ground state isomer in the administered dose: approximately 86% ^197g^Hg in [^197m/g^Hg]HgCl_2_ and 82% ^197g^Hg in [^197m/g^Hg]Hg-Tetrathiol. Combined with its higher relative abundance at time of injection and superior quantification accuracy by gamma counting, particularly in tissues with high uptake, ^197g^Hg was selected as the preferred isotope for further data analysis.

**Fig. 13:**
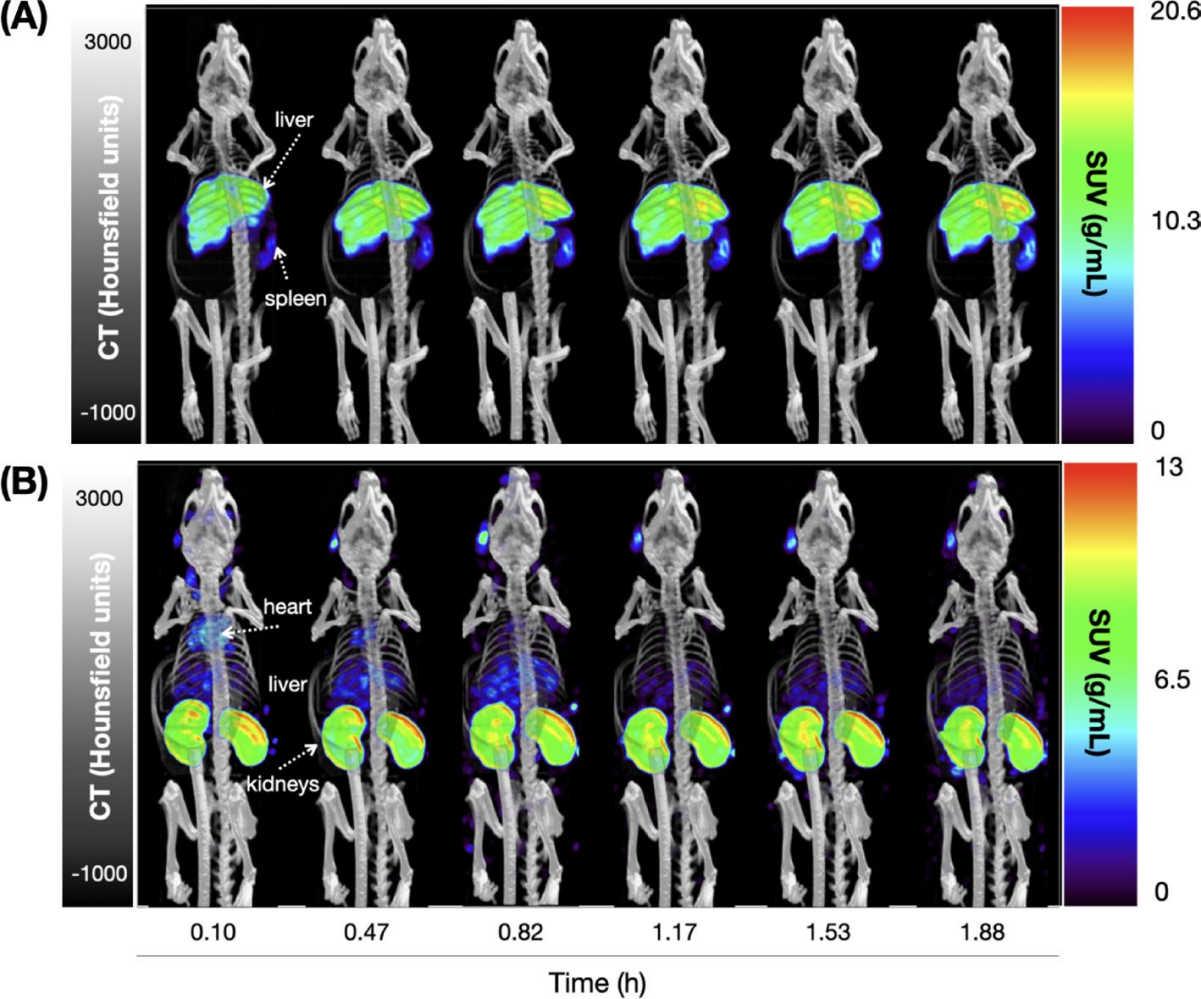
Maximum intensity projection (MIP) images depicting frontal view from a total body SPECT/CT scan obtained 0–2 h following the administration of (**A**) [^197m/g^Hg]Hg-Tetrathiol (0.899 MBq ^197m^Hg + 4.230 MBq ^197g^Hg), with comparison to the total body SPECT/CT scan obtained 2 h following the administration of (**B**) [^197m/g^Hg]HgCl_2_ (0.492 MBq ^197m^Hg + 3.171 MBq ^197g^Hg). Images reconstructed using the 77 keV photopeak

*In vivo SPECT/CT imaging:* One mouse injected with either [^197m/g^Hg]HgCl_2_ or [^197m/g^Hg]Hg-tetrathiol was imaged at multiple time points over ~ 2 h to assess the pharmacodynamic profile of each tracer. All dynamic images were reconstructed using the 77 keV photopeak of ^197g^Hg, which provided the highest image quality due to stronger signal intensity, better detector sensitivity, and lower noise levels (Fig. 13). To illustrate the effect of photopeak selection, a representative scan at ~ 2 h post-injection was also reconstructed using the 134 keV photopeak of ^197m^Hg. The resulting image showed increased noise and reduced signal-to-background contrast, reflecting both the lower amount of ^197m^Hg injected and the reduced detection efficiency at this higher energy (Fig. S14).

For the mouse injected with [^197m/g^Hg]HgCl_2_ (0.492 MBq ^197m^Hg + 3.171 MBq ^197g^Hg), the 77 keV reconstruction clearly outperformed the 134 keV image in terms of signal intensity and noise levels. Although the 134 keV photopeak has a nominally higher emission probability (33.5%), the much lower injected activity of ^197m^Hg, shorter half-life and reduced detector sensitivity at this energy resulted in poorer image quality. These results further support the use of ^197g^Hg and the 77 keV window for quantitative imaging.

The images corroborate the ex vivo biodistribution, with [^197m/g^Hg]HgCl_2_ primarily accumulating in the kidneys while [^197m/g^Hg]Hg-tetrathiol exhibits faster blood clearance and excretion through the reticuloendothelial system (RES) with tracer accumulation primarily in the liver and spleen likely due to the high lipophilicity of the tracer (logD_7.4_ = 0.35 ± 0.1). Standard Uptake Values (SUVs; g/mL) and injected activity per gram (%IA/g) for Hg-197m and Hg-197g images were extracted from the images and are tabulated in Tables S15 and S16. Overall, SUV and %IA/g typically align well when tracer uptake is uniformly distributed in the tissue. However, in the case of kidney uptake for [^197m/g^Hg]HgCl_2_, the activity is predominantly localized in the cortex of the kidneys, so the SUVmean underestimates total activity, while %IA/g derived from voxel-wise analysis will reflect the true uptake.

## Discussion

### H_4_Tetrathiol chelator as an efficient platform for radiomercury radiopharmaceutical development

The chelator studied with Hg^2+^ herein is a commercial pentaerythritol tetrakis(3-mercaptopropionate) acyclic ligand, referred henceforth as H_4_tetrathiol. We used this scaffold as a model to explore and assess the capability of thiol-containing chelators for the rapid, efficient, and stable complexation of [^197m/g^Hg]Hg^2+^ for radiopharmaceutical applications. Thiols are well known to form highly inert complexes with Hg^2+^ in many applications. Most prominently, these Hg^2+^-thiol complexes are seen in vivo, with proteins (metallothionein) and small molecules (glutathione) that contain cysteine. Thiol-containing small molecules have been developed as Hg-poisoning antidotes due to their strong affinity for the metal, allowing them to strip Hg from endogenous proteins and rapidly excrete the metal through the renal system. Although these ligands demonstrate a high affinity for Hg^2+^ ions, they are optimized for rapid excretion and not long-term stability, which is a crucial requirement for chelators incorporated into radiopharmaceuticals. Our selected tetrathiol chelator is a small molecule most commonly used as a precursor in polymer chemistry (Saraswathy et al. 2017; Lazauskas et al. 2019; Huang et al. 2018). However, due to the highly dilute conditions used in radiolabeling, the chelator can be used without polymer formation (Lazauskas et al. 2019). NMR and MS data of ^nat^Hg-tetrathiol suggests the presence of a 1:1 metal-to-ligand complex at the macroscopic scale, though the formation of other species (e.g., 1:2, 2:1 or other metal–ligand complexes) cannot be ruled out without further studies. Computational DFT studies indicate that the 4-coordinate complex is the most thermodynamically favoured configuration. Further investigation into the coordination chemistry is warranted to fully understand the Hg-binding of this chelator, and to unambiguously identify the species present in solution.

Nonetheless, ^197m/g^Hg radiolabeling studies revealed tetrathiol to be a superior chelator in comparison to our previously studied thiacrown macrocycles, exhibiting quantitative or near quantitative radio-mercury incorporation at chelator concentrations as low as 10^–6^ M at physiological temperature (37 °C). RCYs were determined via iTLC using DMSA, a dithiol chelating agent developed for Hg^2+^ poisoning, as a quenching agent. These conditions were optimized in our previous work to ensure negative controls (no chelator added) consistently showed no baseline activity (Rf = 0). Negative controls were again included here to validate results. Nonetheless, DMSA may compete with the tetrathiol chelator, potentially generating mixed Hg-complexes not fully resolved by iTLC. Thus, radioHPLC is necessary to unambiguously identify radiochemical species and assess purity. Unfortunately, radioHPLC of ^197m/g^Hg remains problematic; radiomercury binds strongly to reverse phase columns, causing nearly complete retention of activity within the system. Ongoing efforts are focused on testing alternative mobile phases, gradients, and stationary phases to achieve reproducible and reliable radio-chromatograms with ^197m/g^Hg. Such a development will be critical for advancing the development of ^197m/g^Hg-based radiopharmaceuticals.

Further, the resulting radiometal-complex was inert in vitro when challenged against serum proteins, and excess stable metal ions (ZnCl_2_, FeCl_3_, CuCl_2_, MgCl_2_, and CoCl_2_, or AuCl_3_). However, the ^197m/g^Hg-complex was labile when challenged against ^nat^HgCl_2_. Fortunately, excess Hg^2+^ is not common in the human body; therefore, these results should not pose an issue for in vivo applications. The goal of the assay was to observe the labile nature of Hg^2+^ when radiolabeling with ligands that have a significant affinity to the metal. The assay implied that regardless of the ligand's large affinity to the metal, the labile nature of the Hg^2+^ ion prevailed.

## Isotope selection for in vivo imaging and biodistribution studies

Both ^197m^Hg and ^197g^Hg can reliably resolve structures as small as 1.1 mm. These results are consistent with ^161^Tb SPECT images which resolved rods with diameters ≥ 1.1 mm and outperforms ^226^Ac SPECT images that only resolve rods ≥ 1.3 mm for its 158 keV photopeak and ≥ 1.5 mm for its 230 keV photopeak (Koniar et al. 2022, 2024). Subtle differences in image quality metrics revealed nuanced performance trade-offs between the two isotopes. Specifically, ^197g^Hg provided slightly higher inter-rod contrast and contrast-to-noise ratios for small structures, suggesting improved detectability under low-activity conditions. In contrast, ^197m^Hg showed higher recovery coefficients, indicating more accurate activity quantification, likely due to its reduced photon scatter at the higher energy 134 keV emission energy. The influence of photopeak energy dependence is consistent with previous imaging studies of the resolution phantom with the XUHS collimator.

Despite these technical advantages, ^197g^Hg was ultimately selected for in vivo biodistribution studies based on a combination of practical and biological considerations. Its longer physical half-life (64.1 h vs. 23.8 h for ^197m^Hg) enables imaging at later time points, essential for capturing radiotracer clearance, organ retention, and long-term pharmacokinetics. This flexibility also reduces the need for higher injected doses and simplifies scheduling.

Radiolabeling further favoured ^197g^Hg, as radiopharmaceutical formulations consistently yielded a higher proportion of this isomer (vide supra), resulting in improved counting statistics in both in vivo imaging and ex vivo gamma counting. The 77 keV emissions of ^197g^Hg are also well suited to standard gamma counter settings, enhancing sensitivity and quantitative accuracy in ex vivo tissue measurements.

A common concern with using ^197g^Hg is its isomeric relationship to ^197m^Hg (approximately 91.4% of ^197m^Hg decays into ^197g^Hg over time) creating a coupled decay system. While this could introduce complexity in longitudinal studies, we addressed this challenge directly by developing *HgQuant*, a dedicated Python-based tool that automates Bateman equation modeling. *HgQuant* performs accurate time-resolved decay corrections by integrating initial activities, isotope-specific decay constants, and session timestamps, eliminating manual calculations and user error. By streamlining this process, *HgQuant* not only removes any analytical barrier but also ensures robust, reproducible quantification of both isotopes in phantom and in vivo SPECT datasets. This solution made ^197g^Hg not just viable, but the most practical and powerful choice for our study.

Taken together, these advantages positioned ^197g^Hg as the most robust and practical choice for our in vivo and biodistribution studies. Its favorable physical properties, prominence in radiopharmaceutical formulations, and compatibility with automated time-resolved quantification made it ideally suited to meet the demands of longitudinal imaging in complex biological models.

In moving towards translation, further studies will need to address dosimetry and activity requirements for therapeutic use. While detailed absorbed-dose studies have not yet been performed, the identical chemistry of the isomeric pair is expected to greatly facilitate correlation between imaging and therapy. Toxicity, as with other heavy-metal radiopharmaceuticals, will require preclinical evaluation but is expected to be manageable at tracer-level activities, facilitated by the use of non-carrier added ^197m/g^Hg. Importantly, the use of a mixed-isomeric formulation eliminates the need for complex isotope separation, simplifying preparation and offering opportunities to balance imaging and therapy within a single solution. These considerations delineate a practical roadmap for further development, building on the production feasibility and favorable decay properties already described above.

## Conclusions

The commercially available H_4_Tetrathiol ligand demonstrates strong potential for radiomercury chelation. Radiolabeling with no carrier added [^197m/g^Hg]Hg^2+^ achieved the lowest ligand-to-metal ratio reported for radio-mercury to date. The resulting complex exhibited high in vitro stability against serum proteins, glutathione (GSH), biologically relevant metals (ZnCl_2_, FeCl_3_, CuCl_2_, MgCl_2_ and CoCl_2_) and AuCl_3_, though it showed kinetic lability in the presence of excess stable HgCl_2_. In vivo, the pharmacodynamic profile of [^197m/g^Hg]Hg-Tetrathiol differed from unchelated [^197m/g^Hg]HgCl_2_, indicating complex stability over the study duration.

Despite its high lipophilicity and corresponding liver and spleen uptake, which proved useful for evaluating tracer stability, this property may hinder future use in targeted radiopharmaceuticals, particularly when conjugated to peptides or antibodies. To address this, future studies should explore hydrophilic modifications or linker strategies to improve solubility and reduce off-target hepatic retention.

The resolution and uniformity phantom studies demonstrated an image resolution of ≥ 1.1 mm for both images reconstructed from ^197g^Hg and ^197m^Hg using the XUHS collimator. Additionally, the uniformity phantom yielded half-life values consistent with the known decay constants for each isomer. To our knowledge, this is the first study to conduct a comprehensive phantom-based evaluation of ^197m^Hg and ^197g^Hg, enabling quantitative validation of resolution, recovery coefficients, and decay accuracy. These experiments were essential to generating isotope-specific calibration factors and benchmarking image-based quantification of dual-isotope ^197m/g^Hg systems.

To address the analytical complexity introduced by the isomeric decay relationship between ^197m^Hg and ^197g^Hg, we also developed *HgQuant*, a dedicated Python-based tool that automates Bateman decay correction using time-stamped activity and isotope-specific decay constants. The integration of *HgQuant* with our phantom-calibrated image analysis ensured accurate, reproducible quantification across both phantom and in vivo datasets—minimizing user error and enabling time-resolved activity estimation in longitudinal studies.

Future work should focus on detailed dosimetry, preclinical safety evaluation, and optimization of activity levels to further advance ^197m/g^Hg for clinical application.

Altogether, this work establishes a methodological foundation for dual-isotope ^197m/g^Hg imaging and supports continued investigation of Tetrathiol and other thiol-based ligands as scaffolds for theranostic radiopharmaceutical development.

## Supplementary Information

Below is the link to the electronic supplementary material.

Supplementary Material 1

## Data Availability

The raw data supporting the conclusion of this article is included in part in the supporting information and/or will be made available by the authors, without undue reservation.

## Abbreviations

B3LYP: Becke, 3-parameter, Lee-Yang-Parr
BFC: Bifunctional chelator
CNR: Contrast to noise ratio
D3-BJ: Becke–Johnson damping
DFT: Density functional theory
DMSA: Dimercaptosuccinic acid
DMSO: Dimethylsulfoxide
ECP: Effective core potential
GSH: L-glutathione
H4Tetrathiol: Pentaerythritol tetrakis(3-mercaptopropionate)
HOMO: Highest occupied molecular orbital
HRMS: High resolution mass spectrometry
LUMO: Lowest unoccupied molecular orbital
MAE: Meitner-Auger electron
MIP: Maximum intensity projection
MM: Molecular mechanics
NMR: Nuclear magnetic resonance
NS4-BA: *N*-benzyl-2-(1,4,7,10-tetrathia-13-azacyclopentadecan-13-yl)acetamide
RC: Recovery coefficient
RCY: Radiochemical yield
RES: Reticuloendothelial system
ROI: Region of interest
SDS-PAGE: Sodium Dodecyl Sulphate–PolyAcrylamide Gel Electrophoresis
SD: Stuttgart Dresden
SG: Silica gel
SPECT: Single photon emission computed tomography
SUV: Standard uptake value
TLC: Thin layer chromatography
VOI: Volume of interest
XUHS: Extra ultra-high sensitivity
