# Supplementary material for: Quantitative dual-isotope preclinical SPECT/CT imaging and biodistribution of the mercury-197m/g theranostic pair with [197m/gHg]HgCl2 and a [197m/gHg]Hg-tetrathiol complex as a platform for radiopharmaceutical development
Source: EJNMMI Radiopharm Chem. 2025 Oct 14;10:67. doi: 10.1186/s41181-025-00391-2 (PMC12521730; doi:10.1186/s41181-025-00391-2)
Supplement: Supplementary file 1 — Supplementary Material 1 [file 41181_2025_391_MOESM1_ESM.pdf]

Supporting Information For:

Quantitative dual-isotope preclinical SPECT/CT imaging and biodistribution of the mercury-197m/g theranostic pair with [$^{197m/g}$Hg]$HgCl_2$ and a [$^{197m/g}$Hg]Hg-tetrathiol complex as a platform for radiopharmaceutical development

Parmissa Randhawa[1,2], Cristina Rodríguez-Rodríguez[3,4], Helena Koniar[2,4], Patrick R.W.J. Davey[1,2], Shaohuang Chen[1,2], Valery Radchenko[2,5] and Caterina F. Ramogida[1,2*]

[1] Department of Chemistry, Simon Fraser University, 8888 University Drive, Burnaby, British Columbia, V5A 1S6, Canada.

[2] Life Sciences, TRIUMF, 4004 Wesbrook Mall, Vancouver, British Columbia, V6T 2A3, Canada.

[3]Faculty of Pharmaceutical Sciences, University of British Columbia, 2405 Wesbrook Mall, Vancouver, British Columbia, V6T 1Z3, Canada,

[4]Department of Physics and Astronomy, University of British Columbia, 6224 Agronomy Road, Vancouver, British Columbia, V6T 1Z1, Canada,

[5] Department of Chemistry, University of British Columbia, 2036 Main Mall, Vancouver, British Columbia, V6T 1Z1, Canada

*correspondence: cfr@sfu.ca

Table of Contents

S1. Tetrathiol Ligand and $^{nat}Hg^{2+}$-Complex Characterization 2

S1.1. Tetrathiol ligand NMR characterization 2

S1.2. Hg^{2+} Complex Synthesis and Characterization 5

S1.3. Computational Studies 8

S1.3.1 Thermodynamic calculations and determination of Gibbs free energy (ΔG): 13

S2. HgQuant application 14

S3. Ex Vivo Biodistribution Studies 15

S4. In Vivo SPECT/CT Imaging Studies 19

S1. Tetrathiol Ligand and $^{nat}Hg^{2+}$-Complex Characterization

S1.1. Tetrathiol ligand NMR characterization

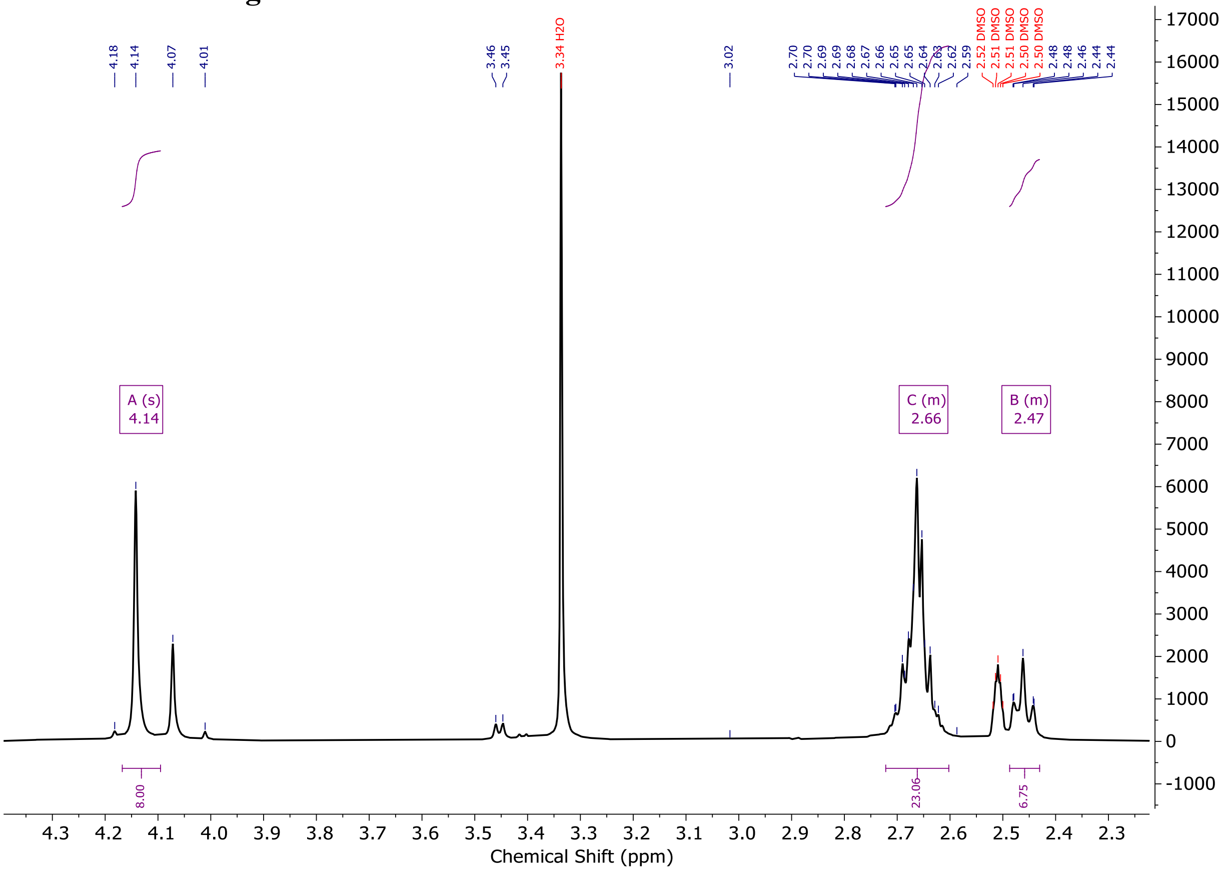

Figure S1. ^{1}H NMR (400 MHz, DMSO-d_6, 25°C): **Tetrathiol**.

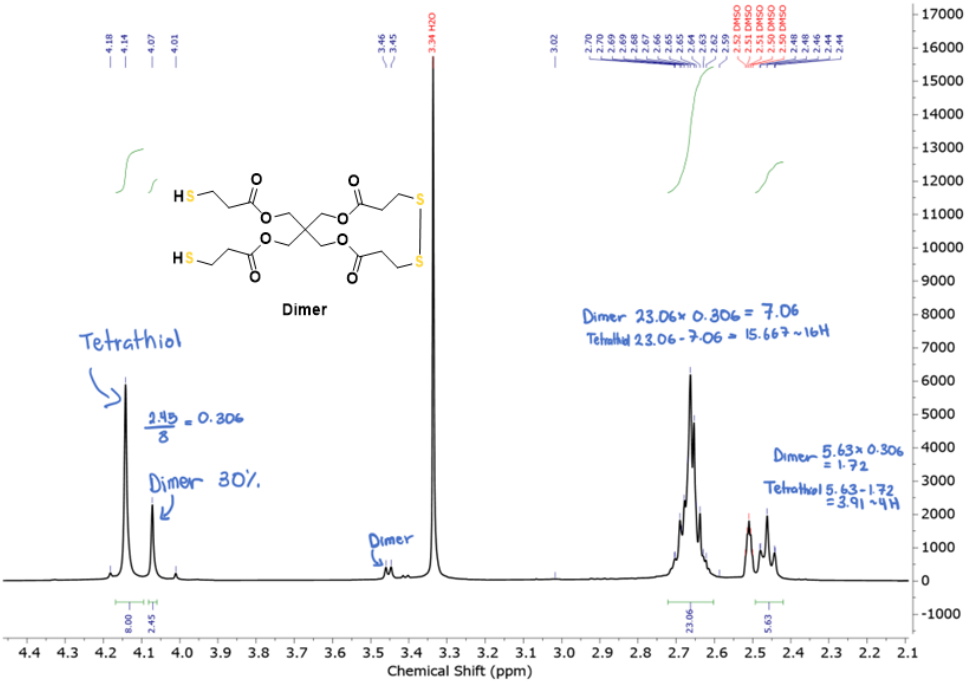

Figure S2. ^{1}H NMR (400 MHz, DMSO-d_6, 25°C): **Tetrathiol** spectrum with dimer peak assignment.

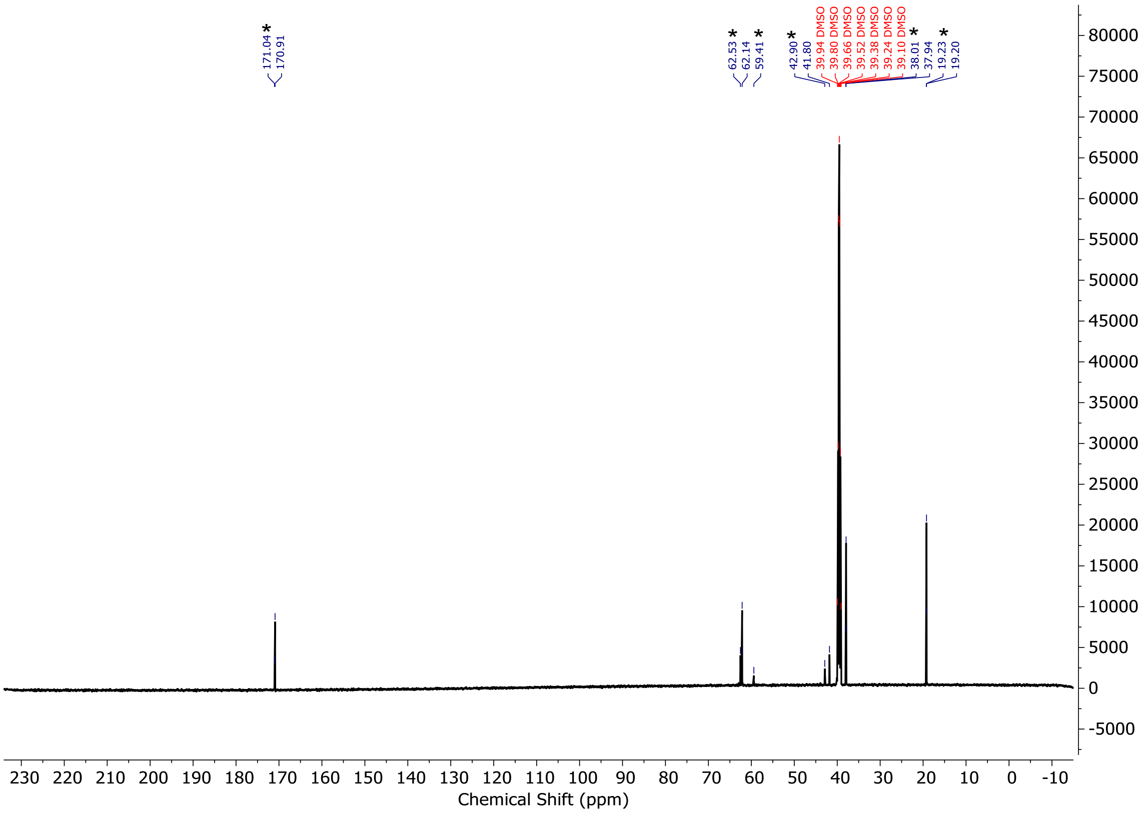

Figure S3. $^{13}C\{^{1}H\}$ NMR (151 MHz, DMSO-d_6, 25°C): **Tetrathiol**, *dimer.

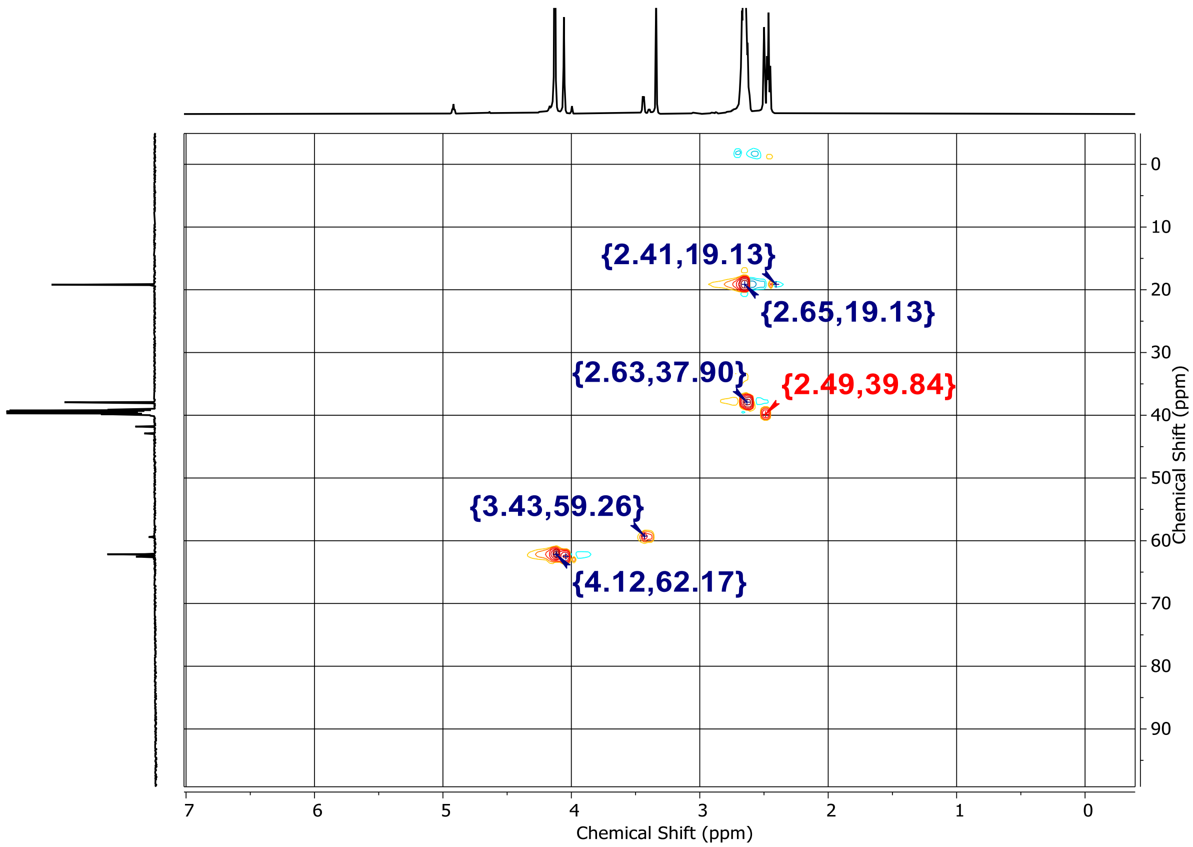

Figure S4. ^{1}H-^{13}C HSQC NMR (600-151 MHz, DMSO-d_6, 25°C): **Tetrathiol**.

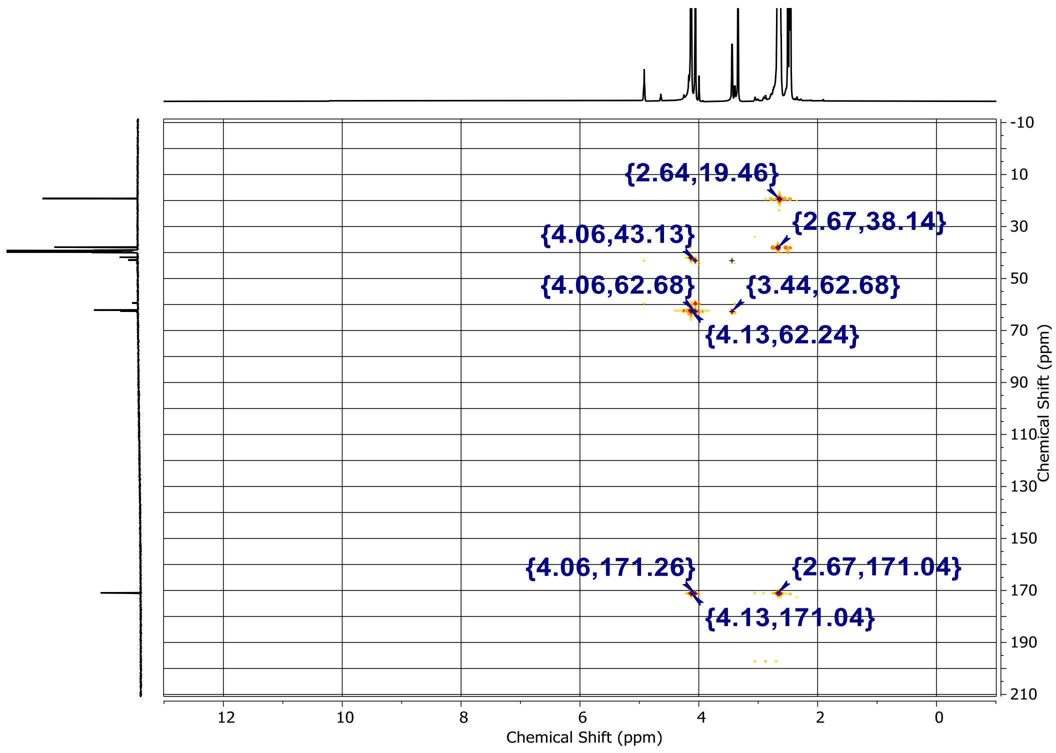

Figure S5. ¹H -¹³C HMBC NMR (600-151 MHz, DMSO-d_6, 25°C): **Tetrathiol**.

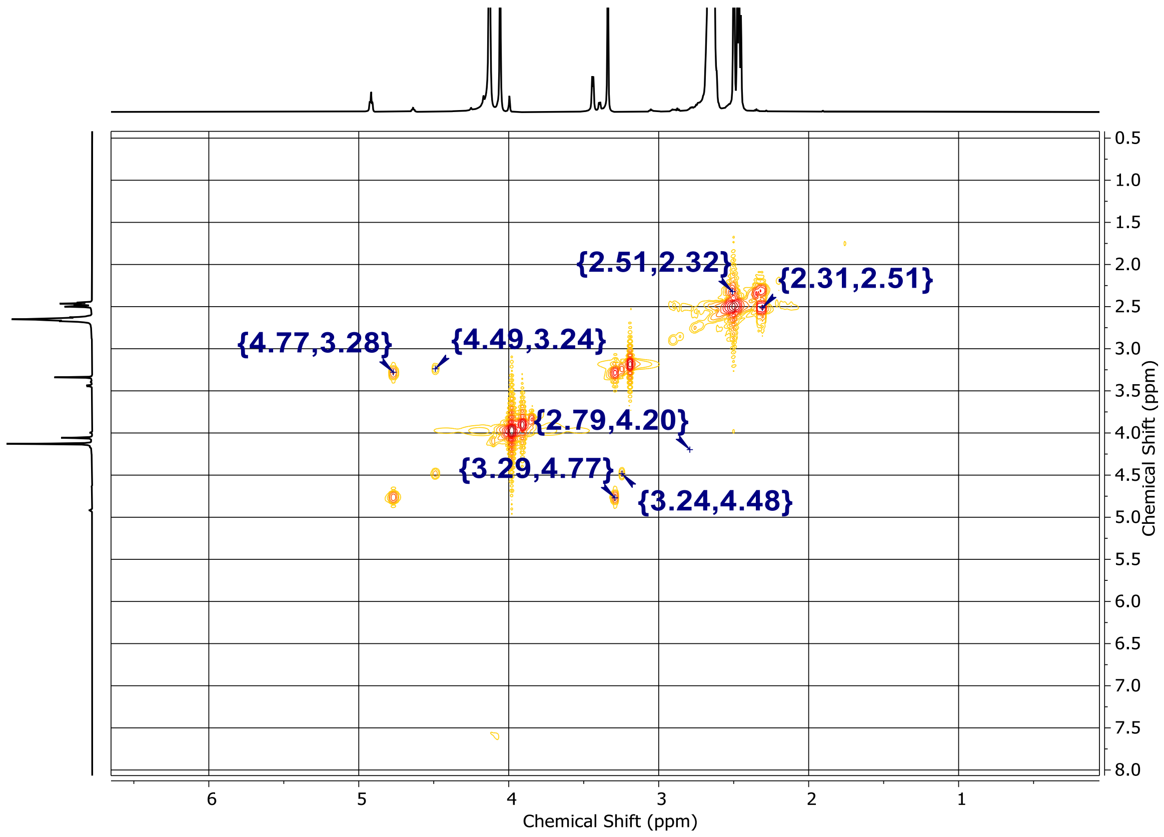

Figure S6. ¹H -¹H COSY NMR (600 MHz, DMSO-d_6, 25°C): **Tetrathiol**.

S1.2. Hg^{2+} Complex Synthesis and Characterization

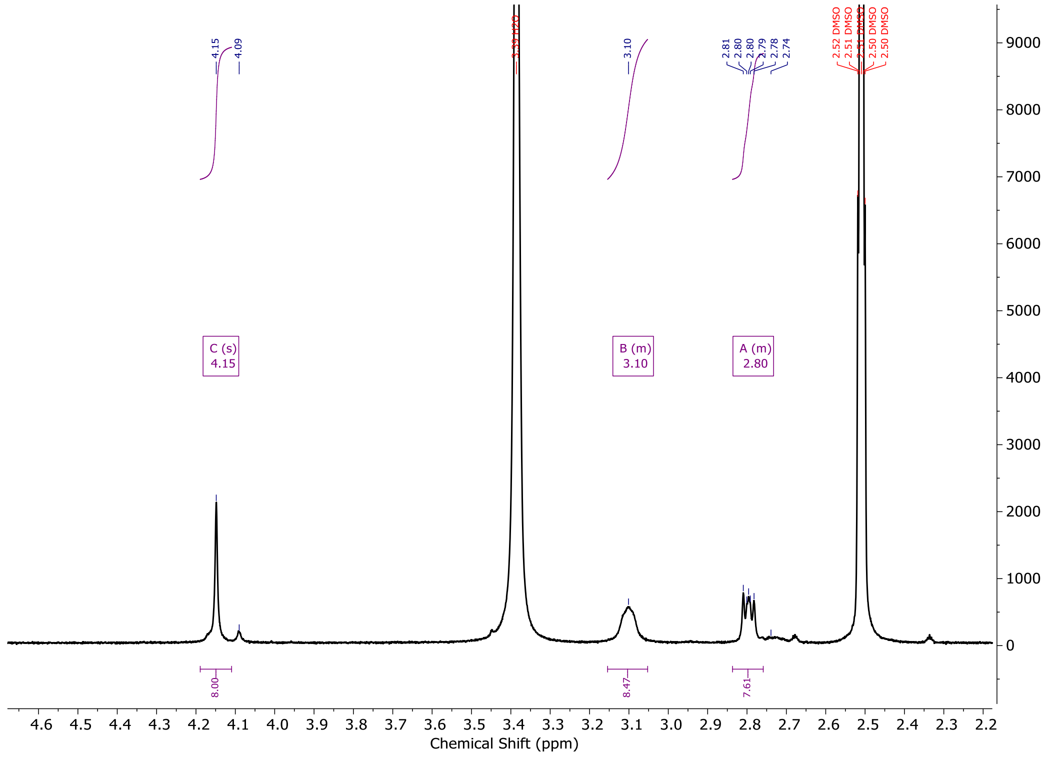

Figure S7. ^{1}H NMR (400 MHz, DMSO-d_6, 25°C): **$[Hg(Tetrathiol)]^{2-}$**.

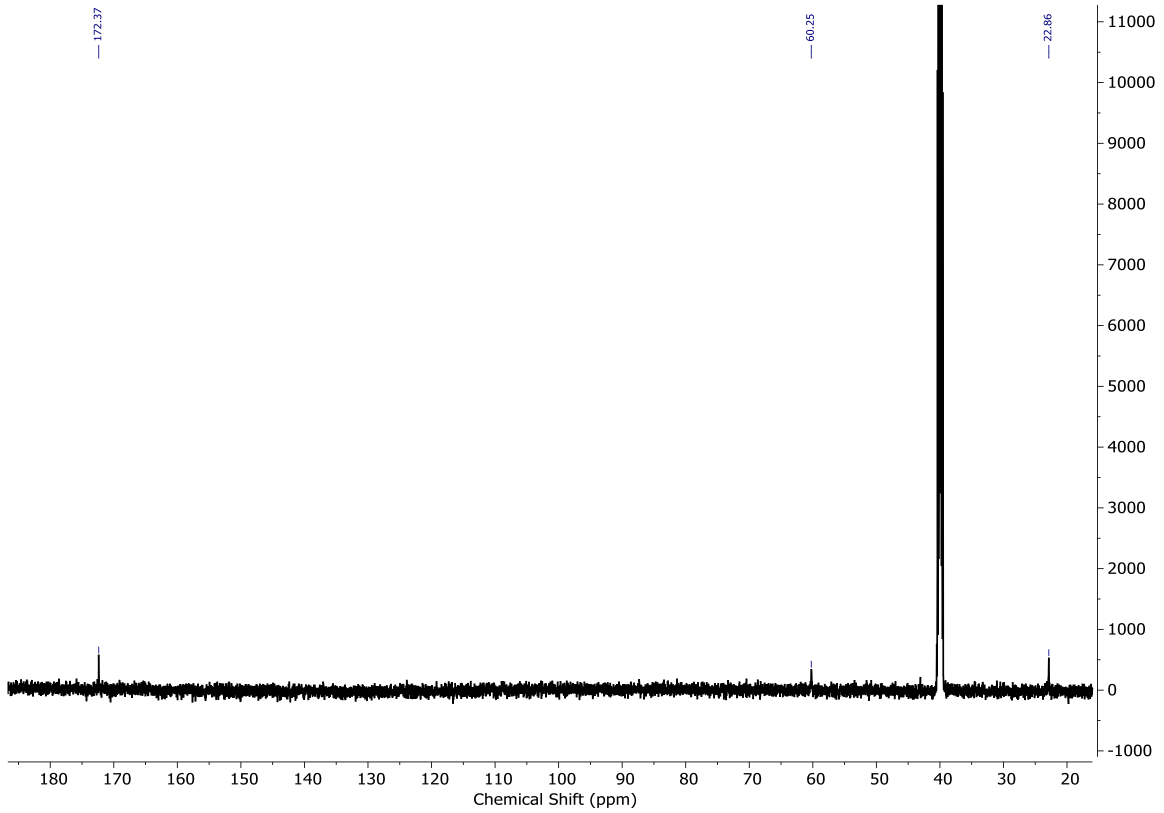

Figure S8. $^{13}C\{^{1}H\}$ NMR (151 MHz, DMSO-d_6, 25°C **$[Hg(Tetrathiol)]^{2-}$**.

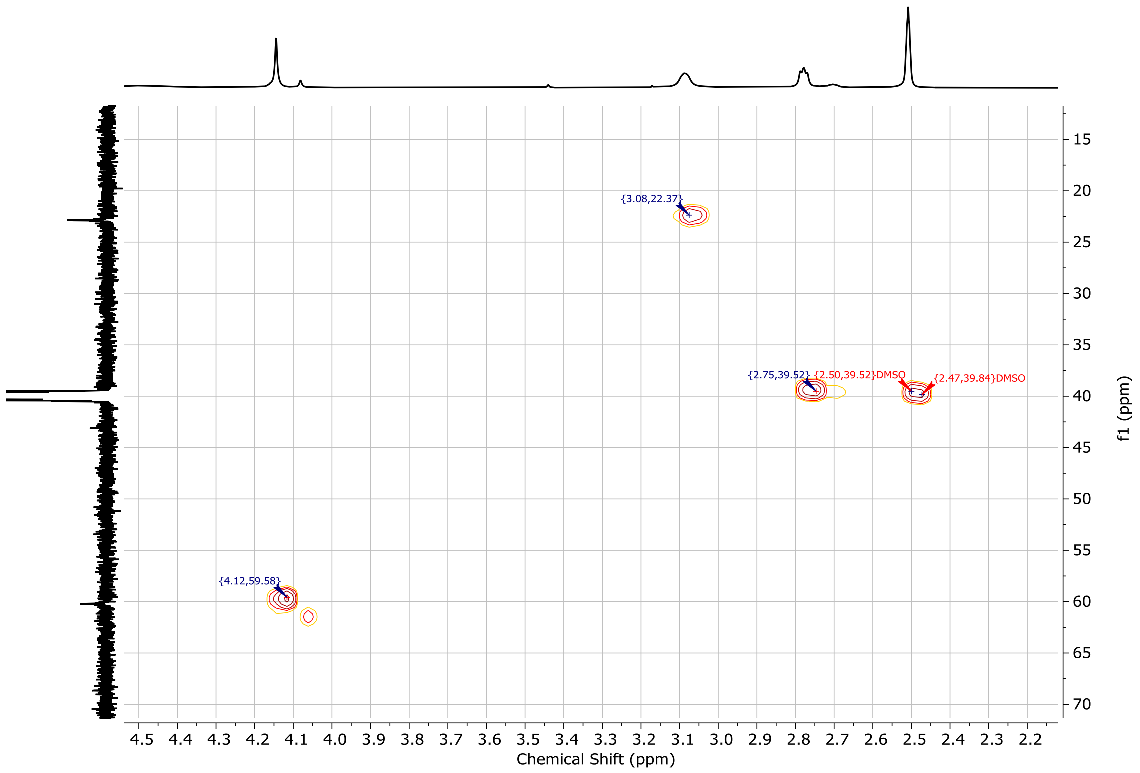

Figure S9. ^{1}H-^{13}C HSQC NMR (600-151 MHz, DMSO-d_6, 25°C): **[Hg(Tetrathiol)]$^{2-}$**.

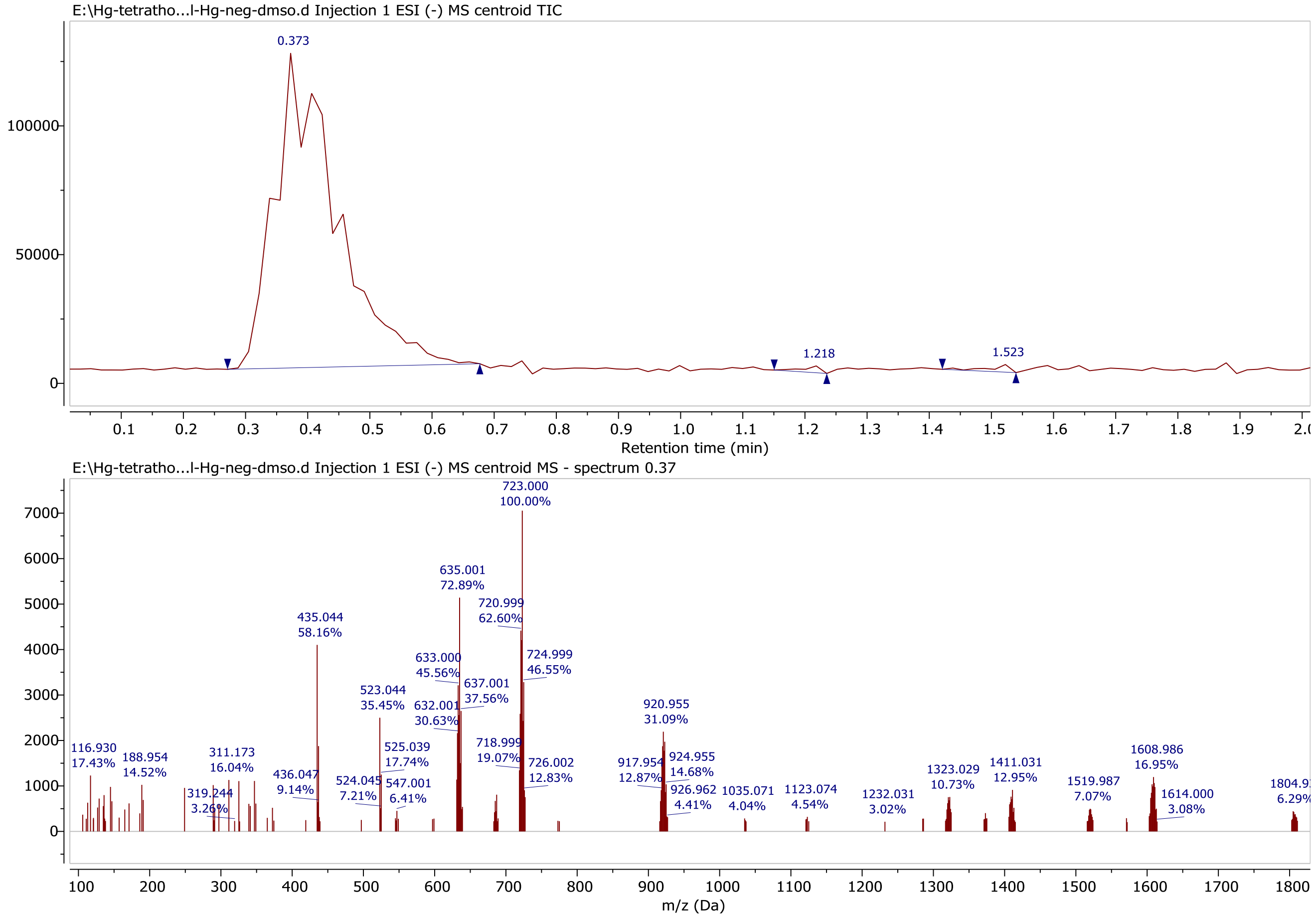

Figure S10. HRMS (ESI negative) spectra of Hg-Tetrathiol complex.

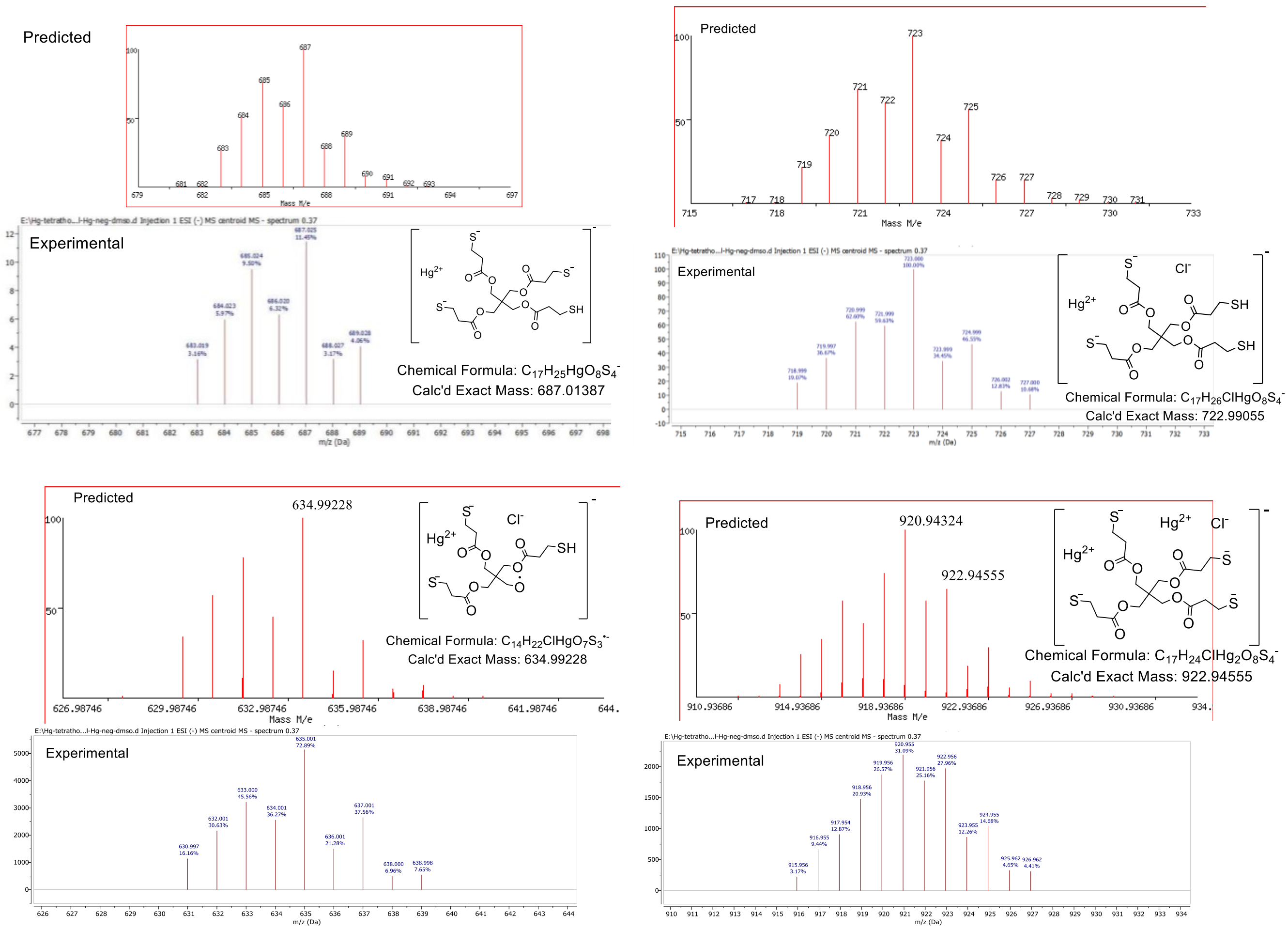

Figure S11. HRMS (ESI negative) spectra of Hg-Tetrathiol complex showing selected main ion peaks stacked with predicted isotope distribution patterns.

S1.3. Computational Studies

Table S1. The 4-coordinate Hg-tetrathiol complex intermolecular distances (Å) and bond angles (°) calculated at the B3LYP-D3/6-311G**/SDD(Hg)/IEFPCM(H_2O) level of theory.

**Interatomic Distances**	**Bond length (Å)**
Hg-S1	2.353
Hg-S2	2.493
Hg-S3	2.591
Hg-S4	2.397
**Interatomic Angles**	**Bond angle (°)**
S1-Hg-S4	124.03
S2-Hg-S3	146.13
S1-Hg-S2	87.63
S1-Hg-S3	89.84
S2-Hg-S4	107.88
S3-Hg-S4	101.44

Table S2. The 4-coordinate Hg-tetrathiol complex Gaussian thermochemistry output.

**Gaussian thermochemistry output**	**Hartree/particle**
Zero-point correction	0.000730
Thermal correction to Energy	0.005732
Thermal correction to Enthalpy	0.006677
Thermal correction to Gibbs Free Energy	-0.037127
Sum of electronic and zero-point energies	-2858.727419
Sum of electronic and thermal energies	-2858.722416
Sum of electronic and thermal enthalpies	-2858.721472
Sum of electronic and thermal free energies	-2858.765276

Table S3. The Hg2-tetrathiol complex intermolecular distances (Å) and bond angles (°) calculated at the B3LYP-D3/6-311G**/SDD(Hg)/IEFPCM(H_2O) level of theory.

**Interatomic Distances**	**Bond length (Å)**
Hg1-S1	2.391
Hg1-S2	2.389
Hg2-S3	2.392
Hg2-S4	2.380
**Interatomic Angles**	**Bond angle (°)**
S1-Hg1-S2	166.49
S3-Hg2-S4	167.98

Table S4. The Hg2-tetrathiol complex Gaussian thermochemistry output.

**Gaussian thermochemistry output**	**Hartree/particle**
Zero-point correction	0.415405
Thermal correction to Energy	0.45019
Thermal correction to Enthalpy	0.451134
Thermal correction to Gibbs Free Energy	0.34358
Sum of electronic and zero-point energies	-2859.220324
Sum of electronic and thermal energies	-2859.185539
Sum of electronic and thermal enthalpies	-2859.184595
Sum of electronic and thermal free energies	-2859.292149

Table S5. The 3-coordinate Hg-tetrathiol complex intermolecular distances (Å) and bond angles (°) calculated at the B3LYP-D3/6-311G**/SDD(Hg)/IEFPCM(H_2O) level of theory.

**Interatomic Distances**	**Bond length (Å)**
Hg-S1	2.474
Hg-S2	2.481
Hg-S3	2.484
Hg-S4	5.853
**Interatomic Angles**	**Bond angle (°)**
S1-Hg-S2	120.65
S1-Hg-S3	110.62
S2-Hg-S3	128.59

Table S6. The 3-coordinate Hg-tetrathiol complex Gaussian thermochemistry output.

**Gaussian thermochemistry output**	**Hartree/particle**
Zero-point correction	0.413097
Thermal correction to Energy	0.446155
Thermal correction to Enthalpy	0.447099
Thermal correction to Gibbs Free Energy	0.345323
Sum of electronic and zero-point energies	-2858.410025
Sum of electronic and thermal energies	-2868.376967
Sum of electronic and thermal enthalpies	-2858.376023
Sum of electronic and thermal free energies	-2858.477799

Table S7. Thermochemistry properties of the Hg^{2+} complexes

**System**	**Δ*G* (kcal/mol)**	**Relative Δ*G* (kcal/mol)**	***E*(HOMO) (eV)**	***E*(LUMO) (eV)**	**HOMO-LUMO gap (eV)**	**Δ*H* (kcal/mol)**	**Relative Δ*H* (kcal/mol)**
4-coordinate $[Hg(\mathbf{L})]^{2-}$	-232.38	0.00	-4.724	-0.470	4.254	-230.65	0.00
3-coordinate $[Hg(\mathbf{L})]^{2-}$	-51.98	180.40	-4.834	-0.172	4.662	-13.88	216.77
$[Hg_2(\mathbf{L})]$	-90.04	142.34	-5.939	-0.483	5.456	4.23	234.88

Table S8. Cartesian coordinates for 4-coordinate [Hg(Tetrathiol)]$^{2-}$

Atom	X	Y	Z
S	1.09878	-0.297671	3.139176
C	0.312593	1.306563	2.770485
C	-0.049999	-3.138888	-2.556747
H	-3.403838	1.455654	-3.006416
C	-4.134429	-0.03381	-0.248221
S	1.203484	-3.135139	0.052294
H	-1.131656	-3.067408	-0.745643
H	-0.369026	-4.621085	-1.016729
C	-1.209262	1.197963	2.802444
H	0.676623	2.12023	3.432302
H	0.656572	1.567879	1.77775
C	-1.905368	2.255477	1.972551
H	-1.529468	0.240247	2.389099
H	-1.531752	1.221621	3.866067
O	-2.580241	3.095528	2.545202
C	-4.321326	-1.396724	0.333428
C	-3.553889	-1.551407	1.657936
O	-1.907879	2.240162	0.603231
H	-5.411384	-1.490969	0.531446
C	-0.199377	-3.526001	-1.086849
C	-1.28291	1.311146	-0.222537
O	1.02407	-1.268415	-3.460825
O	-3.505157	0.13173	-1.44303
C	-1.596424	1.560573	-1.729155
H	-0.223167	1.354696	-0.06693
H	-1.575956	0.285277	0.061346
C	-3.118431	1.370168	-1.936188
C	0.067094	-1.658758	-2.804242
H	-3.654394	-0.684634	2.343825
C	-1.180205	3.019553	-2.130111
H	-4.029439	-2.205785	-0.370189
H	-4.049793	-2.384637	2.199617
C	-0.799699	0.586565	-2.665081
O	-0.859103	-0.762983	-2.328526
H	-1.111447	0.718382	-3.724643
O	0.201999	3.191827	-2.10524
H	-1.508852	3.190949	-3.177977
H	-1.681621	3.788922	-1.503028
C	0.988706	3.271128	-0.997736
H	0.260148	0.895146	-2.638485
O	-4.518186	0.93214	0.398878
O	0.53899	3.69201	0.058582
H	-0.961714	-3.490295	-3.086394
H	0.823193	-3.673235	-2.991286
C	2.420165	2.8079	-1.067871
H	-3.649993	2.193418	-1.408636
S	-1.837356	-2.190781	1.492137
C	2.717049	1.592736	-0.158696
H	2.669974	2.528192	-2.114173
H	3.073873	3.656463	-0.773129

**S**	1.857994	0.06389	-0.7234
**H**	3.803563	1.371696	-0.213061
**H**	2.528567	1.827626	0.900507
**Hg**	0.456826	-1.104097	0.975356

Table S9. Cartesian coordiantes for [Hg_2(Tetrathiol)]

Atom	X	Y	Z
**S**	6.043626	-1.298185	2.441474
**C**	4.747598	0.017137	2.598406
**C**	-3.232018	-1.585177	-0.661642
**S**	-5.780724	-2.783595	-0.975898
**H**	-4.608805	-1.061244	-2.240579
**H**	-3.868436	-2.645775	-2.424629
**C**	3.427962	-0.34249	1.924515
**H**	4.589525	0.168131	3.667113
**H**	5.129655	0.95101	2.190897
**C**	2.44901	0.808182	1.904839
**H**	3.589215	-0.632048	0.881907
**H**	2.954319	-1.202332	2.403296
**O**	2.711912	1.953142	2.193473
**C**	-4.482302	1.94948	0.893491
**C**	-5.863759	2.17776	0.286055
**O**	1.227871	0.396959	1.50457
**H**	-4.327395	0.884145	1.090172
**C**	-4.293304	-1.953324	-1.698681
**C**	0.190128	1.395606	1.38971
**O**	-1.58933	-1.16012	-2.384088
**C**	0.004105	1.885626	-0.055193
**H**	-0.717759	0.906909	1.730596
**H**	0.425983	2.23372	2.044106
**C**	-1.053451	3.007295	-0.049204
**C**	-2.045631	-0.898102	-1.295023
**H**	-6.134292	3.231998	0.360949
**C**	1.266012	2.567502	-0.614315
**H**	-4.374154	2.452143	1.855298
**H**	-5.848984	1.917717	-0.769922
**C**	-0.366591	0.739449	-0.999933
**O**	-1.535436	0.058221	-0.49385
**H**	-0.579449	1.119906	-2.000061
**O**	2.244888	1.558604	-0.928506
**H**	1.032182	3.122616	-1.525047
**H**	1.687599	3.25594	0.118206
**C**	3.328445	1.952032	-1.630497
**H**	0.449742	0.023578	-1.066057
**O**	-3.427579	2.431337	-1.23007
**O**	3.53164	3.099321	-1.949509
**H**	-2.862701	-2.490781	-0.169166
**H**	-3.635281	-0.945437	0.122287
**C**	4.209936	0.767847	-1.940975
**S**	-7.203314	1.220465	1.139226

**C**	5.682284	1.143651	-2.097693
**H**	4.065161	0.028357	-1.153597
**H**	3.834436	0.306815	-2.860479
**S**	6.797603	-0.335474	-2.144012
**H**	5.835473	1.687499	-3.029455
**H**	5.992056	1.796818	-1.282195
**O**	-2.241652	2.644906	0.68063
**H**	-0.658455	3.884367	0.464841
**H**	-1.325731	3.276439	-1.068346
**C**	-3.359182	2.369795	-0.025062
**Hg**	6.574644	-1.013394	0.138949
**Hg**	-6.692055	-0.924036	0.218217

Table S10. Cartesian coordinates for 3-coordinate $[Hg(Tetrathiol)]^{2-}$

Atom	X	Y	Z
**S**	1.09878	-0.297671	3.139176
**C**	0.312593	1.306563	2.770485
**C**	-0.049999	-3.138888	-2.556747
**H**	-3.403838	1.455654	-3.006416
**C**	-4.134429	-0.03381	-0.248221
**S**	1.203484	-3.135139	0.052294
**H**	-1.131656	-3.067408	-0.745643
**H**	-0.369026	-4.621085	-1.016729
**C**	-1.209262	1.197963	2.802444
**H**	0.676623	2.12023	3.432302
**H**	0.656572	1.567879	1.77775
**C**	-1.905368	2.255477	1.972551
**H**	-1.529468	0.240247	2.389099
**H**	-1.531752	1.221621	3.866067
**O**	-2.580241	3.095528	2.545202
**C**	-4.321326	-1.396724	0.333428
**C**	-3.553889	-1.551407	1.657936
**O**	-1.907879	2.240162	0.603231
**H**	-5.411384	-1.490969	0.531446
**C**	-0.199377	-3.526001	-1.086849
**C**	-1.28291	1.311146	-0.222537
**O**	1.02407	-1.268415	-3.460825
**O**	-3.505157	0.13173	-1.44303
**C**	-1.596424	1.560573	-1.729155
**H**	-0.223167	1.354696	-0.06693
**H**	-1.575956	0.285277	0.061346
**C**	-3.118431	1.370168	-1.936188
**C**	0.067094	-1.658758	-2.804242
**H**	-3.654394	-0.684634	2.343825
**C**	-1.180205	3.019553	-2.130111
**H**	-4.029439	-2.205785	-0.370189
**H**	-4.049793	-2.384637	2.199617
**C**	-0.799699	0.586565	-2.665081
**O**	-0.859103	-0.762983	-2.328526
**H**	-1.111447	0.718382	-3.724643

**O**	0.201999	3.191827	-2.10524
**H**	-1.508852	3.190949	-3.177977
**H**	-1.681621	3.788922	-1.503028
**C**	0.988706	3.271128	-0.997736
**H**	0.260148	0.895146	-2.638485
**O**	-4.518186	0.93214	0.398878
**O**	0.53899	3.69201	0.058582
**H**	-0.961714	-3.490295	-3.086394
**H**	0.823193	-3.673235	-2.991286
**C**	2.420165	2.8079	-1.067871
**H**	-3.649993	2.193418	-1.408636
**S**	-1.837356	-2.190781	1.492137
**C**	2.717049	1.592736	-0.158696
**H**	2.669974	2.528192	-2.114173
**H**	3.073873	3.656463	-0.773129
**S**	1.857994	0.06389	-0.7234
**H**	3.803563	1.371696	-0.213061
**H**	2.528567	1.827626	0.900507
**Hg**	0.456826	-1.104097	0.975356

S1.3.1 Thermodynamic calculations and determination of Gibbs free energy (ΔG):

$[Hg(H_2O)_6]^{2+}$ + Tetrathiol^{4-} $\rightleftharpoons$ $[Hg(Tetrathiol)]^{2-}$ + $6H_2O$

$\Delta G_1 = G([Hg(Tetrathiol)]^{2-}) + 6G(H_2O) - G([Hg(H_2O)_6]^{2+}) - G(Tetrathiol^{4-})$ **(S1)**

$2[Hg(H_2O)_6]^{2+}$ + Tetrathiol^{4-} $\rightleftharpoons$ $[Hg_2(Tetrathiol)]$ + $12H_2O$

$\Delta G_2 = G([Hg_2(Tetrathiol)]) + 12G(H_2O) - 2G([Hg(H_2O)_6]^{2+}) - G(Tetrathiol^{4-})$ **(S2)**

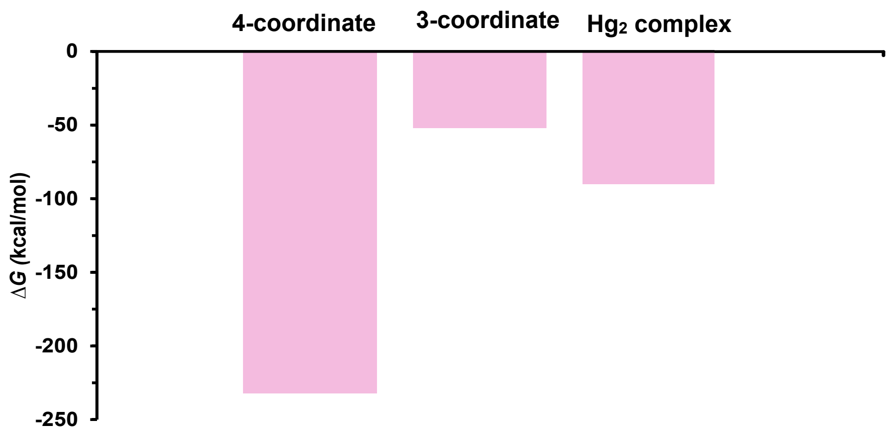

Figure S12. The resulting Gibbs free energies of formation (ΔG) calculated using Equations **S1** and **S2**, for three Hg_x-tetrathiol complexes.

S2. HgQuant application

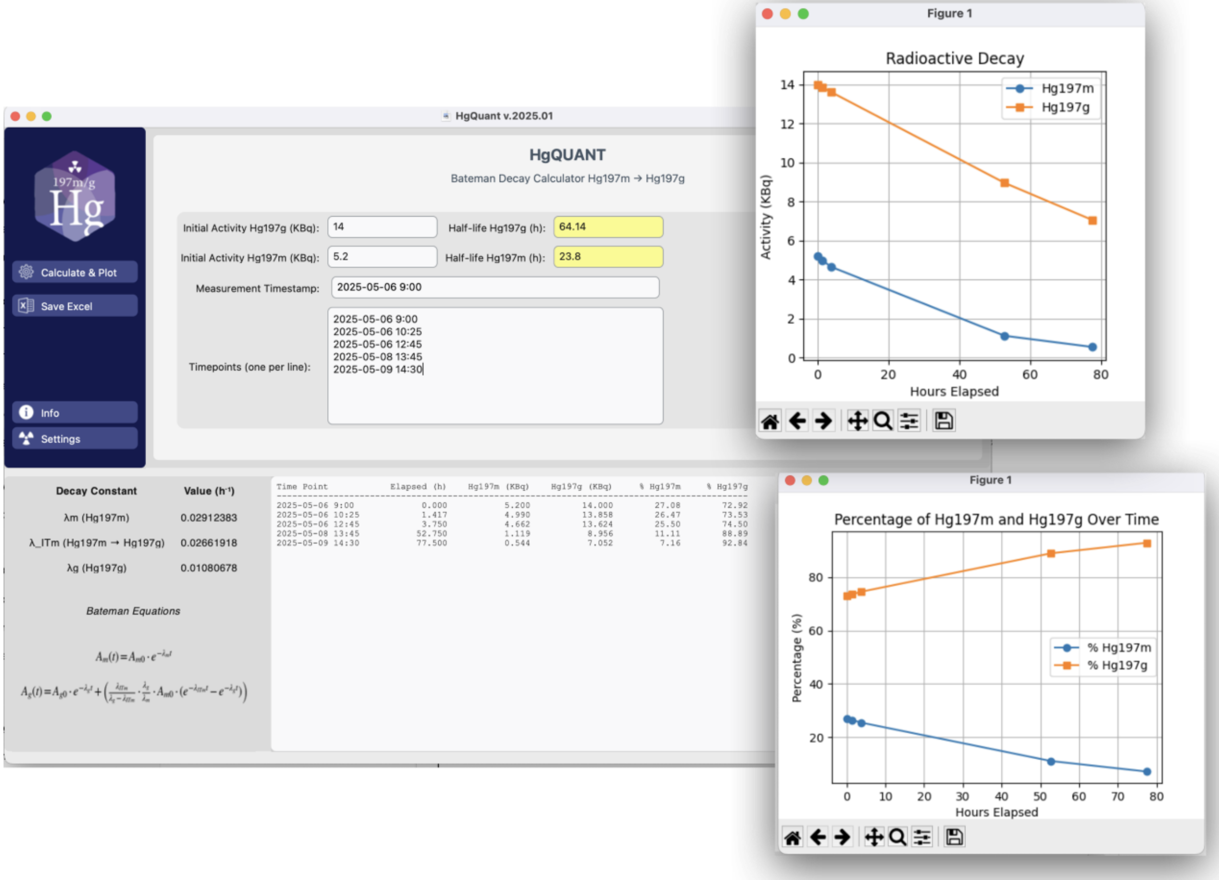

Figure S13. Screenshot of the custom Python-based application *HgQuant*. The tool accepts initial activity values, decay constants, and timepoints, and outputs corrected activities for ^{197m}Hg and ^{197g}Hg using the Bateman equations. This tool was used to standardize decay corrections throughout the imaging and biodistribution experiments in this study.

S3. Ex Vivo Biodistribution Studies

Table S11. Biodistribution results for [^{197m}Hg/^{197g}Hg]$HgCl_2$ at 2 hours post administration in healthy male C57BL/6 mice represented as percentage of injected activity per gram of tissue (%IA/g); organs measured via gamma counter and quantified for metastable (m; 110 – 160 keV gamma window) and ground (g; 55 – 93 keV gamma window) state isomers. Administered activity quantified by HPGe gamma spectroscopy.

	Animal 1 (%IA/g)		Animal 2 (%IA/g)		Animal 3 (%IA/g)		Animal 4 (%IA/g)		Average Hg-197m (%IA/g)		Average Hg-197g (%IA/g)	
**Organ**	**m**	**g**	**m**	**g**	**m**	**g**	**m**	**g**	**Average**	**SD**	**Average**	**SD**
**blood**	8.43	4.25	5.94	3.53	5.50	3.33	4.96	3.00	6.21	1.33	3.53**	0.46
**urine**	8.15	5.10	5.31	3.81	5.63	3.90	5.02	3.49	6.03	1.24	4.08*	0.61
**feces**	1.31	0.96	0.59	0.42	3.93	2.78	1.53	1.01	1.84	1.25	1.29	0.89
**brain**	0.27	0.19	0.15	0.10	0.17	0.11	0.17	0.12	0.19	0.05	0.13	0.03
**tail**	16.30	7.68	7.26	4.62	7.21	4.40	5.98	3.76	9.19	4.14	5.12	1.52
**muscle**	1.38	0.94	0.74	0.52	0.79	0.53	0.81	0.53	0.93	0.26	0.63	0.18
**bone**	3.43	2.26	2.79	1.87	2.67	1.79	2.78	1.85	2.92	0.30	1.94**	0.18
**bladder**	1.81	1.28	4.08	2.73	2.14	1.50	4.20	2.90	3.06	1.09	2.10	0.72
**pancreas**	2.69	1.76	1.94	1.31	2.04	1.39	2.24	1.46	2.23	0.29	1.48**	0.17
**spleen**	8.59	5.78	4.74	3.43	4.18	2.94	5.42	3.58	5.73	1.71	3.93	1.09
**kidneys**	ND [a]	110	ND [a]	107	ND [a]	108	ND [a]	120	ND [a]	ND [a]	111.17	4.99
**liver**	6634.26 [b]	27.68 [b]	10.33	5.13	10.14	4.96	10.31	4.99	10.26	0.08	5.03**	0.08
**heart**	3.85	2.62	2.24	1.60	2.10	1.46	2.14	1.49	2.58	0.73	1.79	0.48
**lungs**	8.68	5.68	4.50	3.11	5.07	3.44	4.53	3.08	5.70	1.74	3.83	1.08
**stomach**	3.64	2.45	2.42	1.70	2.28	1.57	2.56	1.76	2.72	0.54	1.87*	0.34
**small intestine**	6.99	4.46	4.20	2.72	4.50	2.89	3.96	2.62	4.92	1.21	3.17*	0.75
**large intestine**	5.33	3.42	3.94	2.61	3.68	2.41	3.97	2.62	4.23	0.64	2.77**	0.39

[a]ND = not determined due to gamma counter range and artifacts in measuring metastable state photon energy window at extended time points. [b] outlier omitted. Statistical analysis between average %ID/g for Hg-197m and Hg-197g: student unpaired *t*-test: $p < 0.05$ = *; $p < 0.01$ = **; $p < 0.001$ = ***.

Table S12. Biodistribution results for [^{197g}Hg]$HgCl_2$ at 2 hours post administration in healthy male C57BL/6 mice represented as percentage of injected activity per organ (%IA/Organ); organs measured via gamma counter and quantified for ground state isomer (g; 55 – 93 keV gamma window). Administered activity quantified by HPGe gamma spectroscopy.

Organ	Animal 1 (%ID/Organ)	Animal 2 (%ID/Organ)	Animal 3 (%ID/Organ)	Animal 4 (%ID/Organ)	**Average (%ID/Organ)**	**Stdev**
**blood**	7.19	5.88	5.49	4.67	5.81	0.91
**urine**	1.57	0.04	0.63	0.38	0.65	0.57
**feces**	0.04	0.02	0.09	0.07	0.05	0.03
**brain**	0.08	0.04	0.05	0.05	0.05	0.02
**tail**	4.00	2.34	2.23	1.77	2.59	0.85
**muscle**	12.34	6.72	6.84	6.43	8.08	2.46
**bone**	4.96	4.05	3.89	3.74	4.16	0.48
**bladder**	0.10	0.06	0.03	0.07	0.06	0.02
**pancreas**	0.32	0.22	0.25	0.20	0.25	0.04
**spleen**	0.47	0.25	0.22	0.32	0.31	0.10
**kidneys**	51.84	44.76	46.92	49.72	48.31	2.69
**liver**	11.53	6.34	6.61	6.62	7.78	2.17
**heart**	0.39	0.21	0.20	0.21	0.25	0.08
**lungs**	0.87	0.50	0.50	0.46	0.58	0.17
**stomach**	0.29	0.18	0.18	0.17	0.20	0.05
**small intestine**	0.99	0.66	0.62	0.54	0.70	0.17
**large intestine**	0.85	0.60	0.43	0.41	0.57	0.18

Table S13. Biodistribution results for [^{197m}Hg/^{197g}Hg]Hg-tetrathiol at 2 hours post administration in healthy male C57BL/6 mice represented as percentage of injected activity per gram of tissue (%IA/g); organs measured via gamma counter and quantified for metastable (m; 110 – 160 keV gamma window) and ground (g; 55 – 93 keV gamma window) state isomers. Administered activity quantified by HPGe gamma spectroscopy.

	Animal 1 (%IA/g)		Animal 2 (%IA/g)		Animal 3 (%IA/g)		Animal 4 (%IA/g)		Average Hg-197m (%IA/g)		Average Hg-197g (%IA/g)	
**Organ**	**m**	**g**	**m**	**g**	**m**	**g**	**m**	**g**	**Average**	**SD**	**Average**	**SD**
**blood**	0.10	0.09	0.13	0.11	0.15	0.13	0.12	0.10	0.13	0.02	0.11	0.02
**urine**	0.03	0.03	0.03	0.03	0.03	0.03	0.04	0.03	0.033	0.003	0.029	0.004
**feces**	0.04	0.04	0.05	0.05	0.01	0.00	0.01	0.01	0.026	0.018	0.023	0.019
**brain**	0.04	0.03	0.02	0.02	0.04	0.03	0.02	0.02	0.028	0.009	0.025	0.007
**tail**	1.72	1.37	1.39	1.15	0.66	0.56	7.32	5.66	2.77	2.65	2.18	2.03
**muscle**	0.01	0.01	0.02	0.02	0.02	0.02	0.02	0.02	0.017	0.004	0.017	0.003
**bone**	2.09	1.72	1.62	1.37	1.38	1.20	1.62	1.40	1.68	0.26	1.42	0.19
**bladder**	0.05	0.05	0.02	0.04	0.04	0.03	0.07	0.07	0.044	0.017	0.049	0.015
**pancreas**	0.06	0.06	0.04	0.04	0.09	0.08	0.05	0.05	0.056	0.018	0.058	0.016
**spleen**	74.83	44.55	79.95	61.57	108.27	77.82	101.18	73.76	91.06	14.01	64.43*	12.94
**kidneys**	5.53	4.07	2.35	2.02	3.71	3.17	3.03	2.58	3.65	1.18	2.96	0.76
**liver**	ND [a]	85.85	ND [a]	103.98	ND [a]	159.50	ND [a]	460.16	ND [a]	ND [a]	202.37	151.29
**heart**	0.47	0.55	0.59	0.74	0.70	0.82	0.64	0.72	0.60	0.09	0.71	0.10
**lungs**	9.10	7.12	7.04	6.17	9.69	8.45	7.69	6.68	8.38	1.06	7.10	0.85
**stomach**	0.19	0.16	0.37	0.32	1.02	0.89	0.12	0.12	0.42	0.35	0.37	0.31
**small intestine**	0.16	0.14	0.08	0.07	0.10	0.09	0.15	0.13	0.12	0.03	0.11	0.03
**large intestine**	0.14	0.12	0.05	0.05	0.12	0.10	0.05	0.04	0.091	0.040	0.079	0.035

[a]ND = not determined due to gamma counter range and artifacts in measuring metastable state photon energy window at extended time points. Statistical analysis between average %ID/g for Hg-197m and Hg-197g: student unpaired *t*-test: $p < 0.05$ = *; $p < 0.01$ = **; $p < 0.001$ = ***.

Table S14. Biodistribution results for [^{197g}Hg]Hg-Tetrathiol at 2 hours post administration in healthy male C57BL/6 mice represented as percentage of injected activity per organ (%IA/Organ); organs measured via gamma counter and quantified for ground state isomer (g; 55 – 93 keV gamma window). Administered activity quantified by HPGe gamma spectroscopy.

Organ	Animal 1 (%IA/Organ)	Animal 2 (%IA/Organ)	Animal 3 (%IA/Organ)	Animal 4 (%IA/Organ)	Average (%IA/Organ)	SD
**blood**	0.13	0.17	0.18	0.15	0.16	0.02
**urine**	0.00	0.00	0.00	0.00	0.00	0.00
**feces**	0.00	0.00	0.00	0.00	0.00	0.00
**brain**	0.01	0.01	0.01	0.01	0.01	0.00
**tail**	0.69	0.56	0.27	2.67	1.05	0.95
**muscle**	0.14	0.22	0.21	0.20	0.19	0.03
**bone**	3.25	2.68	2.26	2.62	2.70	0.35
**bladder**	0.00	0.00	0.00	0.00	0.00	0.00
**pancreas**	0.01	0.01	0.01	0.01	0.01	0.00
**spleen**	3.22	4.01	4.84	4.62	4.17	0.63
**kidneys**	1.54	0.82	1.16	1.00	1.13	0.26
**liver**	110.52	133.87	131.25	107.31	120.74	11.91
**heart**	0.07	0.09	0.09	0.09	0.09	0.01
**lungs**	0.95	0.87	1.12	0.93	0.97	0.09
**stomach**	0.02	0.04	0.09	0.01	0.04	0.03
**small intestine**	0.03	0.02	0.02	0.02	0.02	0.01
**large intestine**	0.02	0.01	0.02	0.01	0.02	0.01

S4. In Vivo SPECT/CT Imaging Studies

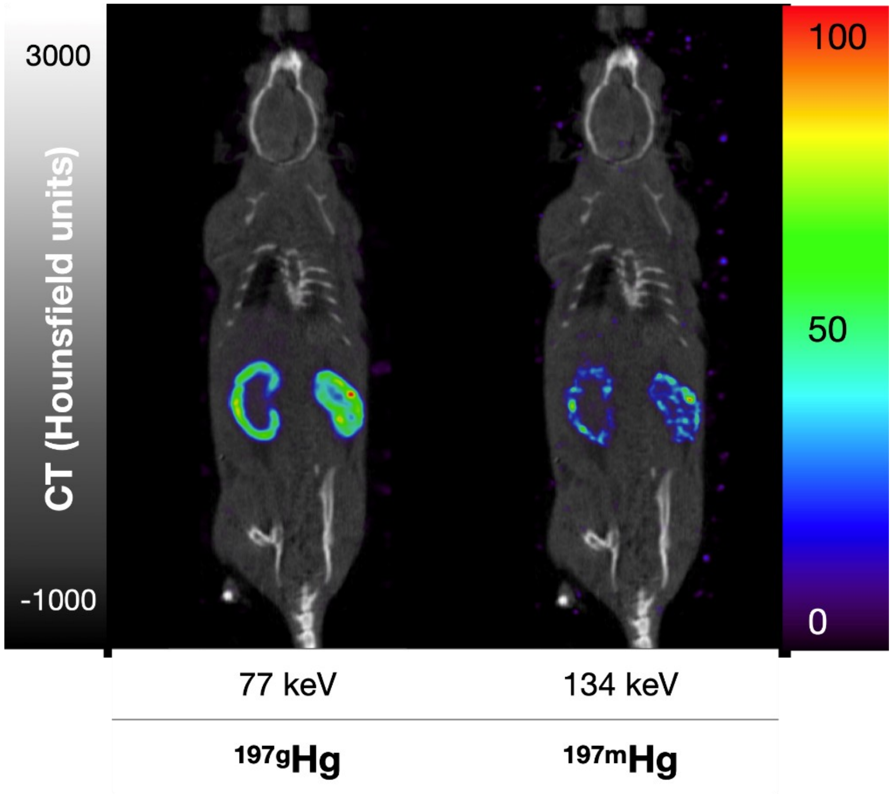

Figure S14. Fused coronal SPECT/CT images of a mouse injected with **[$^{197m/g}$Hg]HgCl$_2$** (0.492 MBq ^{197m}Hg; 3.171 MBq ^{197g}Hg) 1.88 hours post injection. Images reconstructed with the 77 keV window (left) captures emissions from ^{197g}Hg, while the 134 keV window (right) corresponds to ^{197m}Hg.

Table S15. Standard Uptake Value (SUV) and %IA/g values for kidneys, heart, liver, bone, and spleen following injection of 'free' $^{197m/g}$**HgCl₂**, using reconstructions at 77 keV and 134 keV, compared to results obtained from ex vivo biodistribution (BioD, %IA/g) at last time point (~ 2h).

Time		KIDNEYS						BLOOD					
		SUV (g/mL)		%IA/g*		BioD (%IA/g)		SUV (g/mL)		%IA/g		BioD (%IA/g)	
min	Hours	77 keV	134 keV	77 keV	134 keV	Hg-197g	Hg-197m	77 keV	134 keV	77 keV	134 keV	Hg-197g	Hg-197m
6.0	0.10	9.85118	9.74113	56%	60%			4.43868	4.36218	14.10%	13.86%		
28.0	0.47	11.12478	14.15808	63%	87%			2.76176	2.80564	8.77%	8.91%		
49.0	0.82	14.85987	14.76241	84%	89%			2.10395	2.16948	6.68%	6.89%		
70.0	1.17	15.79821	15.97419	89%	96%			1.69271	2.25807	5.38%	7.17%		
92.0	1.53	16.81403	18.69148	94%	111%			1.56310	1.28630	4.97%	4.09%		
113.0	1.88	16.64232	20.10124	93%	118%	109%	-	1.49994	1.36675	4.76%	4.34%	4.25%	8.43%

Time		LIVER						BONE					
		SUV (g/mL)		%IA/g		BioD (%IA/g)		SUV (g/mL)		%IA/g		BioD (%IA/g)	
min	Hours	77 keV	134 keV	77 keV	134 keV	Hg-197g	Hg-197m	77 keV	134 keV	77 keV	134 keV	Hg-197g	Hg-197m
6.0	0.10	2.59395	2.86434	8.24%	9.10%			0.66852	0.79163	1.61%	1.90%		
28.0	0.47	2.51508	2.93720	7.99%	9.33%			0.60826	0.45273	1.46%	1.09%		
49.0	0.82	2.42897	2.12357	7.72%	6.75%			0.54390	0.71122	1.31%	1.71%		
70.0	1.17	2.43116	2.64527	7.72%	8.40%			0.49674	0.76093	1.19%	1.83%		
92.0	1.53	2.31598	1.74186	7.36%	5.53%			0.59002	0.58419	1.42%	1.40%		
113.0	1.88	2.43564	2.34176	7.74%	7.44%	27.68%	-	0.89512	0.50896	2.15%	1.22%	2.26%	3.43%

Time		SPLEEN					
		SUV (g/mL)		*%IA/g*		*BioD (%IA/g)*	
min	Hours	77 keV	134 keV	77 keV	134 keV	Hg-197g	Hg-197m
6.0	0.10	2.33244	4.09950	7.41%	13.02%		
28.0	0.47	1.76797	3.11738	5.62%	9.90%		
49.0	0.82	1.55797	1.81086	4.95%	5.75%		
70.0	1.17	1.42673	1.76313	4.53%	5.60%		
92.0	1.53	1.46475	1.86245	4.65%	5.92%		
113.0	1.88	1.89681	2.95089	6.03%	9.37%	5.78%	8.59%

Table S16. Standard Uptake Value (SUV) and %IA/g values for kidneys, heart, liver, bone, and spleen following injection of **$^{197m/g}$Hg-Tetrathiol**, using reconstructions at 77 keV and 134 keV, compared to results obtained from ex vivo biodistribution (BioD, %IA/g) at last time point (~ 2h).

Time		KIDNEYS						BLOOD					
		SUV (g/mL)		%IA/g*		BioD (%IA/g)		SUV (g/mL)		%IA/g		BioD (%IA/g)	
min	Hours	77 keV	134 keV	77 keV	134 keV	Hg-197g	Hg-197m	77 keV	134 keV	77 keV	134 keV	Hg-197g	Hg-197m
6.0	0.10	0.58804	0.47192	3.71%	3.56%			0.19513	0.15602	0.73%	0.58%		
28.0	0.47	0.66734	0.44617	5.60%	3.36%			0.18666	0.10100	0.70%	0.38%		
49.0	0.82	0.73037	0.56030	6.15%	4.25%			0.22334	0.09399	0.84%	0.35%		
70.0	1.17	0.69040	0.51031	5.82%	3.85%			0.17253	0.17784	0.65%	0.67%		
92.0	1.53	0.70515	0.54796	5.94%	4.13%			0.16589	0.13571	0.62%	0.51%		
113.0	1.88	0.68953	0.45830	5.81%	3.46%	4.07%	5.53%	0.16762	0.09628	0.63%	0.36%	0.09%	0.10%

Time		LIVER						BONE					
		SUV (g/mL)		%IA/g		BioD (%IA/g)		SUV (g/mL)		%IA/g		BioD (%IA/g)	
min	Hours	77 keV	134 keV	77 keV	134 keV	Hg-197g	Hg-197m	77 keV	134 keV	77 keV	134 keV	Hg-197g	Hg-197m
6.0	0.10	15.57810	14.00901	58.32%	52.44%			0.42103	0.32877	1.58%	1.23%		
28.0	0.47	16.79487	14.99573	62.87%	56.14%			0.41977	0.18525	1.57%	0.69%		
49.0	0.82	16.63067	15.30028	62.26%	57.28%			0.47955	0.41000	1.80%	1.53%		
70.0	1.17	16.47068	15.14801	61.66%	56.71%			0.40419	0.21980	1.51%	0.82%		
92.0	1.53	16.50857	15.37946	61.80%	57.58%			0.50524	0.39589	1.89%	1.48%		
113.0	1.88	16.65172	15.74491	62.34%	58.94%	85.85%	-	0.46313	0.39794	1.73%	1.49%	1.72%	2.09%

Time		SPLEEN					
		SUV (g/mL)		%IA/g		BioD (%IA/g)	
min	Hours	77 keV	134 keV	77 keV	134 keV	Hg-197g	Hg-197m
6.0	0.10	8.05430	7.30905	30.15%	27.36%		
28.0	0.47	8.33319	8.21421	31.20%	30.75%		
49.0	0.82	8.79952	7.25015	32.94%	27.14%		
70.0	1.17	8.54783	8.76885	32.00%	32.83%		
92.0	1.53	8.87530	8.48575	33.23%	31.77%		
113.0	1.88	8.59743	8.52722	32.19%	31.92%	44.55%	74.83%
